# Postbiotics From *Lactobacillus Johnsonii* Activates Gut Innate Immunity to Mitigate Alcohol‐Associated Liver Disease

**DOI:** 10.1002/advs.202405781

**Published:** 2024-11-22

**Authors:** Ruopeng Yin, Tao Wang, Jingzu Sun, Huanqin Dai, Yuting Zhang, Ningning Liu, Hongwei Liu

**Affiliations:** ^1^ State Key Laboratory of Mycology Institute of Microbiology Chinese Academy of Sciences Beijing 100101 China; ^2^ Medical School University of Chinese Academy of Sciences Beijing 100049 China; ^3^ CAS Key Laboratory of Pathogenic Microbiology and Immunology Institute of Microbiology Chinese Academy of Sciences Beijing 100101 China

**Keywords:** alcoholic‐associated liver disease, gut microbiota, innate immunity, *Lactobacillus johnsonii*, postbiotics

## Abstract

Prolonged alcohol consumption disrupts the gut microbiota and the immune system, contributing to the pathogenesis of alcohol‐associated liver disease (ALD). Probiotic‐postbiotic intervention strategies can effectively relieve ALD by maintaining gut homeostasis. Herein, the efficacy of heat‐killed *Lactobacillus johnsonii* (HKLJ) in mitigating alcoholic liver damage is demonstrated in mouse models of ALD. The gut‐liver axis is identified as a pivotal pathway for the protective effects of *L. johnsonii* against ALD. Specifically, HKLJ is found to upregulate the expression of intestinal lysozymes, thereby enhancing the production of immunoregulatory substances from gut bacteria, which subsequently activated the Nucleotide‐binding oligomerization domain 2 (NOD2)‐interleukin (IL‐23)‐IL‐22 innate immune axis. The elevated IL‐22 upregulated the antimicrobial peptide synthesis to maintain intestinal homeostasis and moreover activated the Signal transducer and activator of Transcription3 (STAT3) pathway in the liver to facilitate the repair of hepatic injuries. The heat‐killed *L. johnsonii* provoked immunity helps correct the gut microbiota dysbiosis, specifically by reversing the reduction of butyrate‐producing bacteria (such as *Faecalibaculum rodentium*) and the expansion of opportunistic pathogens (such as *Helicobacter* sp. and *Pichia kudriavzevii*) induced by ethanol. The findings provide novel insights into the gut microbiota‐liver axis that may be leveraged to enhance the treatment of ALD.

## Graphical abstract

The postbiotics derived from *L. johnsonii* confer protective effects against ALD by activating NOD2‐mediated gut immunity. This process involves the enhancement of the NOD2‐lysozyme loop and an increase in the population of IL‐22‐producing group 3 innate lymphoid cells. The NOD2‐lysozyme loop facilitates the production of immunoregulatory substances from postbiotics, which subsequently activate the NOD2‐IL‐23‐IL‐22 immune axis. The elevated levels of IL‐22 serve a dual purpose: they activate the STAT3 pathway in the liver, thereby promoting the repair of hepatic injuries associated with ALD, and they stimulate the secretion of antimicrobial peptides in the gut. This secretion is crucial for maintaining gut homeostasis in the context of ethanol‐induced intestinal damage, as it helps to prevent the reduction of butyrate‐producing commensals, such as *F. rodentium*, and the expansion of opportunistic pathogens, including *Helicobacter* sp. and *P. kudriavzevii*. Images are created using BioRender.com.

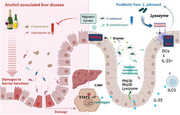

## 1 Introduction

Long‐term alcohol abuse and the resulting alcohol‐associated liver disease (ALD) are recognized as the major contributors to approximately one‐quarter of liver disease‐related deaths worldwide.^[^ ^1^, ^2^ ^]^ Excessive alcohol intake impairs the gut barrier function and immune homeostasis in patients with ALD which leads to the release of microbial toxins or the translocation of pathogens from the gut into the liver, exacerbating ALD.^[^ ^3^, ^4^, ^5^ ^]^ Inspired by these mechanisms mediated via the gut‐liver axis, intervention strategies such as fecal microbiota transplantation (FMT), phage therapy, and the oral supplementation of prebiotics, probiotics, and postbiotics are being used as therapeutic regimens for liver diseases by improving gut barrier function and immune homeostasis.^[^ ^6^, ^7^, ^8^, ^9^ ^]^

The currently available live microbial formulations have shown marginal or highly variable in vivo efficacies in patients.^[^ ^10^, ^11^ ^]^ In contrast to live probiotics, inactivated microorganisms and/or their components (called postbiotics) show more advantages in good stability, safety, and efficacy repeatability. Accumulating investigations on postbiotics have paved the way to unlocking the substances and mechanisms underlying the beneficial effects of gut microbiota on animals and human beings. For example, peptidoglycan fragments produced by commensal *Enterococcus* have been shown to enhance immunotherapy and host defense,^[^ ^12^, ^13^ ^]^ polysaccharide A from the cell wall of specific *Bacteroides* modulates anti‐inflammatory T cell populations,^[^ ^14^, ^15^ ^]^ and surface polysaccharides of *Bifidobacterium* attenuate inflammation by enhancing intestinal regulatory T cell production.^[^ ^16^ ^]^ In studies of ALD, a flagellin from *Roseburia intestinalis* produced an anti‐ALD effect by activating the Toll‐like receptors (TLR) 5 receptor to restore gut barrier function, and postbiotic nanoparticles derived from *Lactobacillus rhamnosus* GG alleviated ALD symptoms via regulation of intestinal microRNA194‐FXR signaling.^[^ ^17^ ^]^ These results suggest that gut microbial postbiotics are promising for the prevention and treatment of ALD by interacting with host intestinal cells, especially immune cells.

Metagenomic analysis revealed a relatively high abundance of *Lactobacillus* species in the gut of healthy individuals.^[^ ^18^ ^]^ *Lactobacillus* species and strains have been shown to have diverse benefits in enhancing host immunity through distinctive modes of action.^[^ ^19^ ^]^ Among them, *Lactobacillus johnsonii*, a commonly detected probiotic in the intestines of healthy hosts possesses important bioactivities in enhancing the efficacy of immunotherapy, improving gut barrier functions, and reducing fatty liver disease.^[^ ^20^, ^21^, ^22^ ^]^ Interestingly, inactivated *L. johnsonii* was demonstrated to protect against both sepsis‐mediated liver injury and acute alcohol‐induced liver injury.^[^ ^23^, ^24^ ^]^ However, the protective mechanisms of inactivated *L. johnsonii* have yet to be elucidated.

In this work, the anti‐ALD effect of gut commensal *L. johnsonii* and its postbiotics was demonstrated in two ALD mouse models. The protective effects of *L. johnsonii* postbiotics were mechanistically linked to the activation of the NOD2‐IL‐22 signaling pathway in intestinal immune cells and the upregulation of the IL‐22‐regulated STAT3 pathway in the liver. Moreover, supplementation with *L. johnsonii* postbiotics helped maintain gut microbiota homeostasis under long‐term ethanol feeding, suppressing the ethanol‐induced overgrowth of opportunistic gut pathogens.

## 2 Results

### 2.1 *L. Johnsonii* Protects Against Liver Injury and Maintains Gut Integrity in ALD

**Figure 1:**
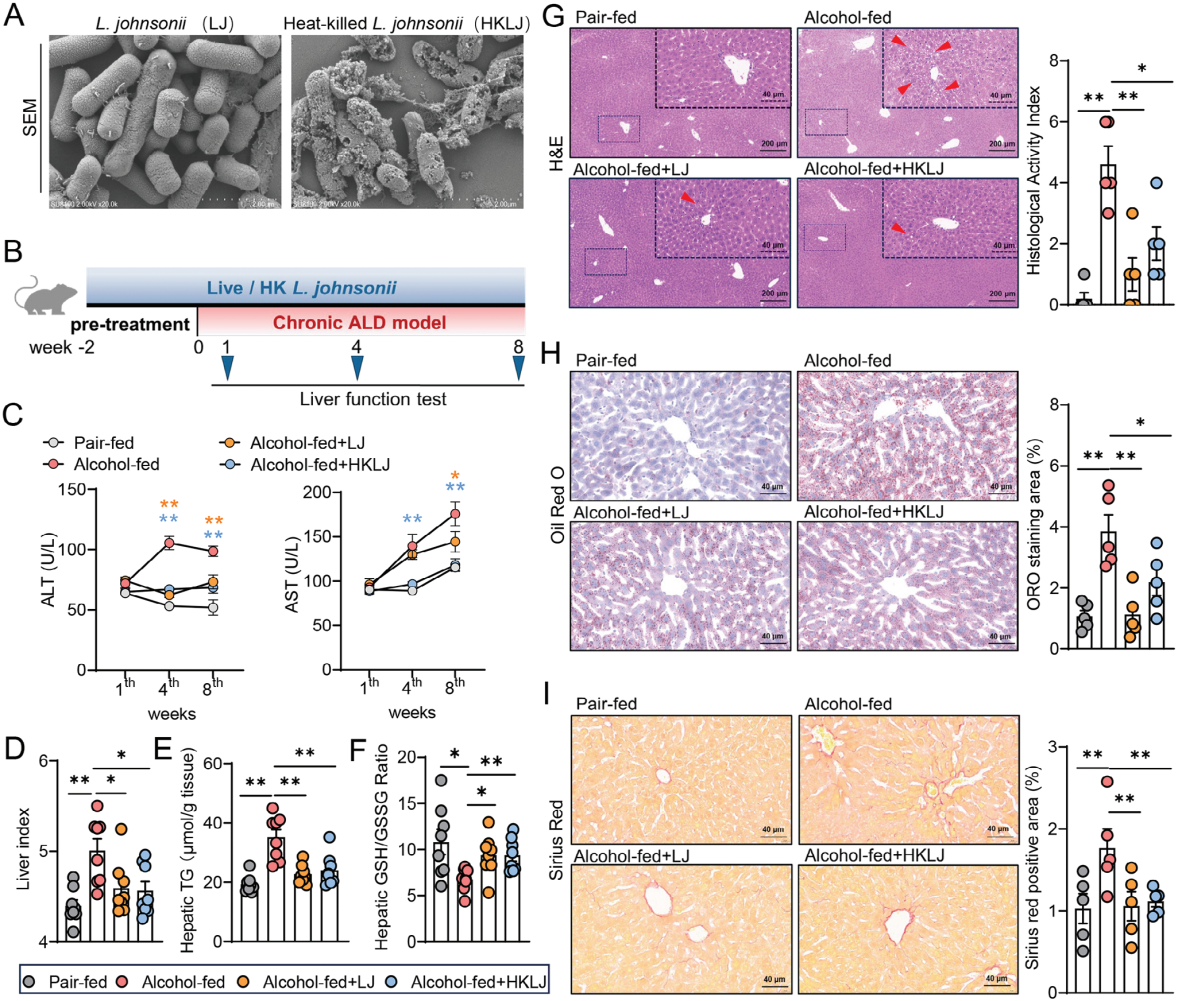
*L. johnsonii* and heat‐killed strain attenuate liver injury. A) Representative SEM images of *L. johnsonii* (LJ) and heat‐killed strains (HKLJ). B) Schematic diagrams of chronic alcohol feeding‐induced ALD mouse model with treatment. C) Serum levels of ALT and AST at weeks 1, 4, and 8 during the intervention. D) Liver‐to‐body weight ratio at the time of sacrifice. E) Triglyceride levels in liver tissue. F) Ratio of GSH and GSSG contents in liver tissue. G) Representative H&E staining images of liver sections and quantitative analysis of liver histologic scores. H) Staining of mouse liver sections for oil red O and quantitative analysis. I) Staining of mouse liver sections for Sirius red and quantitative analysis. Each dot represents data from one animal (biological replicate). Biological replicate numbers in each group are as follows: (Figure 1C, D, E, F) *n* = 8, (Figure 1G, H, I) *n* = 5. Statistical significance was determined using one‐way analysis of variance, and data represent the mean ± s.e.m., unless otherwise indicated. Statistically significant differences are shown with asterisks as follows: ^*^ *p* values < 0.05; ^**^ *p* values < 0.01; ns, no significant differences. SEM: scanning electron microscope; ALT: alanine aminotransferase; AST: aspartate aminotransferase; GSH: reduced glutathione; GSSG: oxidized glutathione; H&E: hematoxylin and eosin.

Fresh *L. johnsonii* (LJ) was pasteurized to generate heat‐killed *L. johnsonii* (HKLJ). Scanning electron microscope (SEM) revealed that HKLJ (right) had broken cell walls according to its micro‐morphology (**Figure** **1**A; Figure S1, Supporting Information). To investigate the protective effect of LJ on ALD injuries, mice fed a normal diet were pretreated with either live LJ (2 × 10^8^ CFUs/day) or HKLJ for two weeks and then fed a Lieber‐DeCarli ethanol liquid diet in a chronic manner while being continuously administrated LJ or HKLJ at the same dosage (Figure 1B). Pair‐fed mice and ethanol‐fed mice were used as negative controls and positive controls, respectively. After six weeks of feeding with ethanol, 33.3% (4 out of 12) of mice died in the ethanol‐fed group, and the surviving mice showed a 10.6% reduction in body weight compared to that at the start of chronic feeding. Supplementation of ethanol‐fed mice with LJ and HKLJ suppressed body weight loss and protected the mice from death (Figure S2A,B, Supporting Information). Both LJ and HKLJ administration alleviated liver injury, as evidenced by significantly decreased alanine aminotransferase (ALT) and aspartate aminotransferase (AST) levels, respectively, in comparison with those in the ethanol‐fed model group (Figure 1C). Importantly, HKLJ more potently reduced AST level than LJ. In addition, supplementation with LJ and HKLJ alleviated the ethanol‐induced increase in the liver index and hepatic triglyceride content and recovered hepatic antioxidant potency as reflected by a 38.8% and 38.5% increase in the ratio of reduced glutathione (GSH) to oxidized glutathione (GSSG), respectively (Figure 1D–F). Histopathological analysis revealed that mice fed ethanol exhibited severe hepatic steatosis (indicated by red arrows), inflammatory cell infiltration, lipid deposition, and fibrosis. Notably, both the LJ and HKLJ interventions significantly attenuated these pathological changes (Figure 1G–I). Further qPCR assays showed that supplementation with LJ and HKLJ down‐regulated the hepatic gene expression of inflammation‐related cytokines including interleukin (IL)‐1β, tumor necrosis factor‐alpha (TNFα), and inducible nitric oxide synthase (iNOS), as well as lipid synthesis rate‐limiting enzymes, such as fatty acid synthetase (FAS) and acetyl‐CoA carboxylase 1 (ACC1) (Figure S2C, Supporting Information). HKLJ has the same effect as LJ in lowering the levels of inflammation‐related cytokines. Immunofluorescence staining of F4/80‐positive hepatic macrophages further supported the reduction in hepatic inflammation in the HKLJ‐treated group (Figure S2D, Supporting Information). The protective effect of HKLJ against ALD was also verified in a chronic plus‐binge alcohol feeding model (NIAAA model), as shown by significant reductions in the liver index, and levels of ALT and AST (Figure S3, Supporting Information). These findings suggest that pasteurization may enhance the beneficial effects of LJ on ALD injuries.

**Figure 2:**
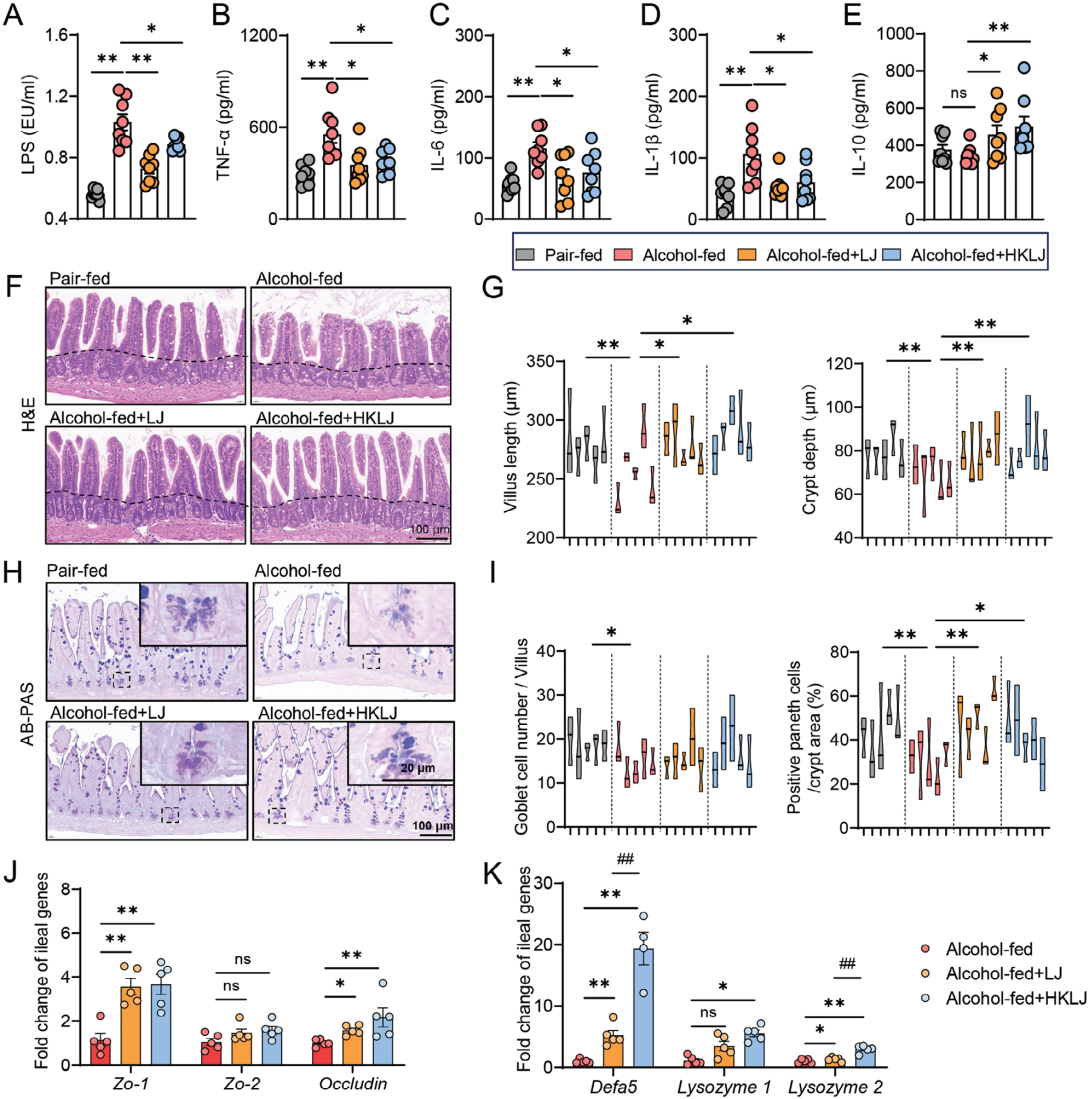
*L. johnsonii* and heat‐killed strains maintain intestinal integrity. A) LPS levels in serum. B) Levels of TNF‐α in serum. C) Levels of IL‐6 in serum. D) Levels of IL‐1β in serum. E) Levels of IL‐10 in serum. F) Representative images of H&E staining of mouse ileum. G) Measurements of villi and crypts in the mouse ileum. H) Representative images of AB‐PAS staining of mouse ileum. I) Measurement of goblet cells and Paneth cells in mouse ileum. J) Ileal mRNA expression levels of *Zo‐1*, *Zo‐2*, and *Occludin*, normalized to *Gapdh*. K) Ileal mRNA expression levels of *Defa5*, *Lysozyme1*, and *Lysozyme2*, normalized to *Gapdh*. Each violin plot representing one animal, and at least 3 villi/crypts were counted per animal (Figure 2G,I). Each dot represents data from individual biological replicate. Biological replicate numbers in each group are as follows: (Figure 2A–E) *n* = 8, (Figure 2J–K) *n* = 4‐5. Statistical significance was determined using one‐way analysis of variance, and data represent the mean ± s.e.m., unless otherwise indicated. Statistically significant differences are shown with asterisks as follows: ^*^ *p* values < 0.05; ^**^ *p* values < 0.01; ns, no significant differences. AB‐PAS: Alcian blue‐periodic acid Schiff; *Gapdh*: glyceraldehyde‐3‐phosphate dehydrogenase.

Alcohol consumption impairs gut barrier functions, which consequently increases circulating endotoxemia and aggravates alcoholic steatohepatitis.^[^ ^25^ ^]^ In the current work, the LJ or HKLJ treatments significantly reversed the negative effects of ethanol feeding, as indicated by a notable increase in the levels of lipopolysaccharide (LPS) and pro‐inflammatory cytokines (TNFα, IL‐6, and IL‐1β) in the circulation of mice (**Figure** **2**A–D). Notably, a 32.3% and 42.5% increase in anti‐inflammatory cytokine (IL‐10) concentrations was observed in the LJ or HKLJ‐treated group, respectively (Figure 2E). As indicated by H&E staining and Alcian blue & periodic acid‐Schiff (AB‐PAS) staining, supplementation with LJ or HKLJ resulted in a recovery of the ileal villus length, the crypt depth (divided by a dotted line between villi and crypts), and the number of Paneth cells in the intestinal crypts compared with ALD controls (Figure 2F–I). Consistent with the intestinal pathological observation, supplementation with LJ or HKLJ alleviated gut barrier dysfunction, as indicated by tight junctions (*Zo‐1*, *Zo‐2*, and *Occludin*) and gut defense molecules (*Lysozyme‐1*, *Lysozyme‐2*, and *Defensin‐5*) in the qPCR assay (Figure 2J,K). Taken together, the above data supported that LJ and HKLJ supplementation restored ethanol‐disrupted gut barrier function.

### 2.2 Heat‐killed *L. Johnsonii* Protects Against Alcohol‐Induced Gut Microbiota Dysbiosis

**Figure 3:**
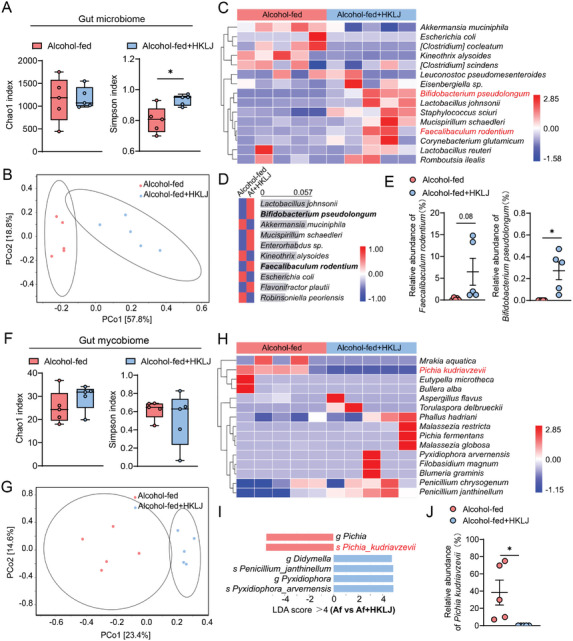
Heat‐killed *L. johnsonii* changes gut bacterial and fungal composition. A) Chao1 index and Simpson index (alpha diversity) of the gut bacterial composition. B) PCoA (beta diversity) plot of gut bacterial composition generated using unweighted Unifrac distance. C) Heatmap for the gut bacterial composition in species taxonomic level. D) Based on the Random Forest analysis, the top 10 species in terms of importance scores for differences between groups were ranked with decreasing scores from top to bottom. E) The relative abundance of *Faecalibaculum rodentium* and *Bifidobacterium pseudolongum* in the gut. F) Chao1 index and Simpson index (alpha diversity) of the gut fungal composition. G) PCoA (beta diversity) plot of gut fungal composition generated using Jaccard distance. H) Heatmap for the gut bacterial composition in species taxonomic level. I) LDA score representing the enriched taxon in the gut mycobiome between two groups. Only LDA scores (>4) are shown. Red indicates enriched taxa in the alcohol‐fed group; blue indicates enriched taxa in the HKLJ group. J) The relative abundance of *Pichia kudriavzevii* in the gut. Each dot represents data from individual biological replicate. Five biological replicate numbers in each group. Statistical significance was determined using two‐tailed Mann‐Whitney U test. Data represent the mean ± s.e.m. For box‐and‐whiskers plots, the center line represents the median, the bounds of the box represent quartiles and the whiskers represent min to max. Statistically significant differences are shown with asterisks as follows: ^*^ *p* values < 0.05; ^**^ *p* values < 0.01; ns, no significant differences. PCoA: Principal co‐ordinates analysis.

Ethanol‐induced impairment of gut integrity is linked to gut microbiota dysbiosis in individuals with ALD.^[^ ^26^ ^]^ Given that supplementation with the postbiotics (HKLJ) rescued ethanol‐disrupted gut barrier functions and promoted the secretion of antimicrobial peptides, we performed 16S ribosomal RNA and ITS gene sequencing of cecum contents to detect changes in the gut microbiome, respectively. HKLJ supplementation significantly increased the bacterial richness according to the Simpson's index compared with that in the ethanol‐fed group (**Figure** **3**A) and greatly altered the bacterial composition according to the beta diversity determined via PCoA (weighted UniFrac distance) (Figure 3B; Figure S4A, Supporting Information). Linear discriminant analysis of effect size (LEfSe) was used to detect dominant differences in the predominance of bacterial communities in the HKLJ‐treated group. At the genus level, LEfSe analysis revealed specific enrichment of the genera *Psychrobacter*, *Pseudomonas*, *Eggerthella*, and *Pseudobutyrivibrio* in the HKLJ‐treated group, comparing with the dominance of *Helicobacter* and *Muribaculum* in the alcohol‐fed group, which was further confirmed by relative abundance analysis of the genera (Figure S4B–D, Supporting Information). At the species level, random forest analysis indicated an increased proportion of *Bifidobacterium pseudolongum*, *Mucispirillum schaedleri*, *Enterorhabdus* sp., *Faecalibaculum rodentium*, and *Flavonifractor plautii* in the HKLJ‐supplemented group (Figure 3C,D). Therein, we found that in the gut of mice treated with HKLJ, the abundance of *F. rodentium* was ≈30‐fold greater than that in the ALD controls, and *B. pseudolongum*, which was not detected in ALD controls, significantly expanded (Figure 3E).

**Figure 4:**
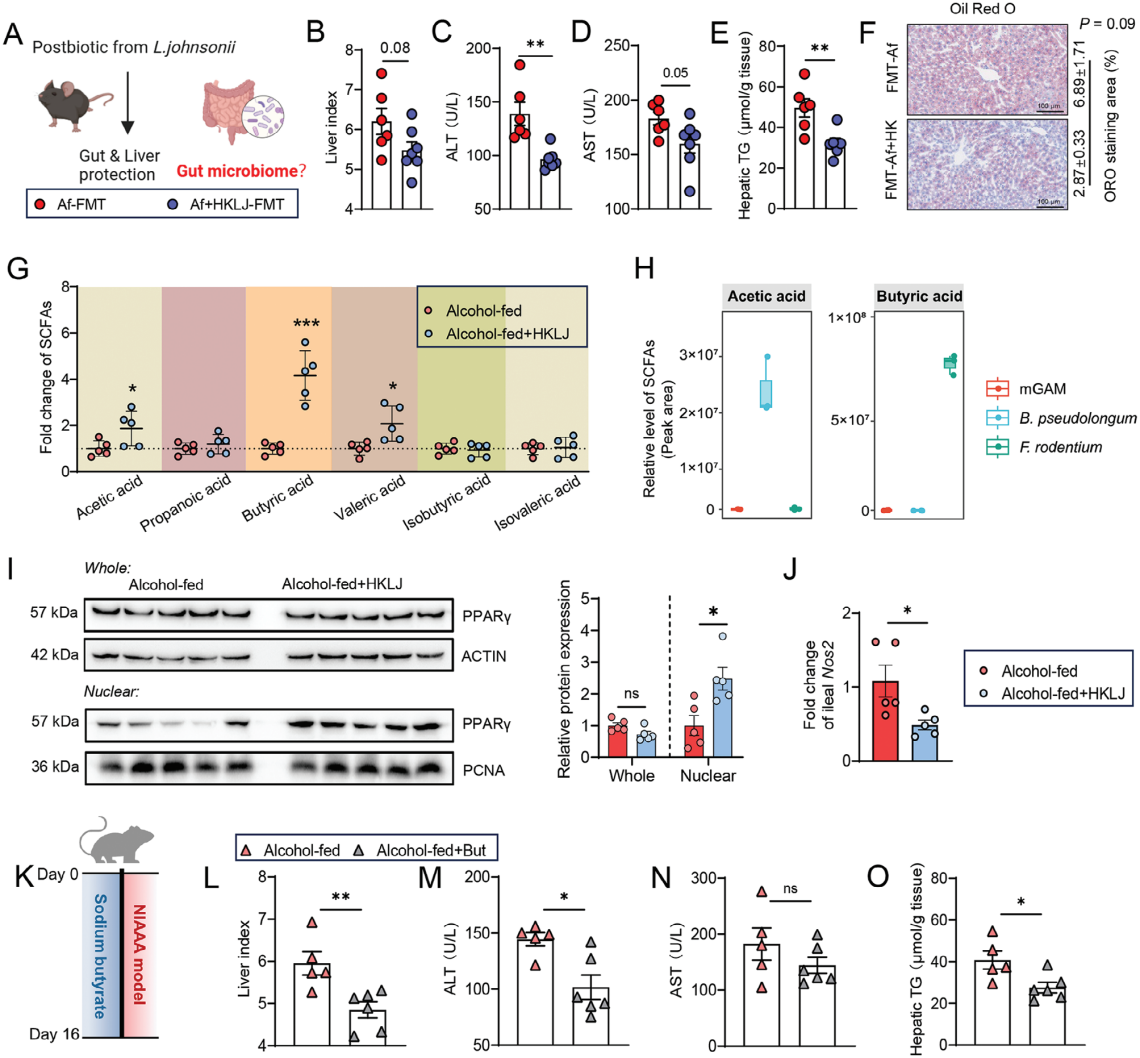
Microbial butyric acid‐mediated intestinal PPARγ helps to suppress ALD. A) Schematic diagrams of Figure 4 (created by BioRender.com). B) Liver‐to‐body weight ratio at the time of sacrifice. C) Serum levels of ALT. D) Serum levels of AST. E) Triglyceride levels in liver tissue. F) Staining of liver sections for oil red O and quantitative analysis. The physiological & biochemical indices and histopathology in (B–F) were measured using serum and liver from fecal microbiota transplant recipient mice from the NIAAA model. G) The relative levels of SCFAs in the fecal samples. H) The levels of acetic acid and butyric acid in the cultured supernatant of *F. rodentium* and *B. pseudolongum*. I) The protein expression of PPARγ in the intestinal epithelium lysate and the cell nucleus lysate by western blot, normalized to β‐actin or PCNA (nucleus). J) Ileal mRNA expression levels of *Nos2*, normalized to *Gapdh*. K) Schematic diagrams of NIAAA mouse model with butyrate treatment. L) Liver‐to‐body weight ratio at the time of sacrifice. M) Serum levels of ALT. N) Serum levels of AST. O) Triglyceride levels in liver tissue. The physiological & biochemical indices in (L–O) were measured using serum and liver from NIAAA model mouse with sodium butyrate treatment. Each dot represents data from individual biological replicate. Biological replicate numbers in each group are as follows: (Figure 4B–E) *n* = 6‐7, (Figure 4F) *n* = 3‐4, (Figure 4G,I,J) *n* = 5, (Figure 4H) *n* = 3, (Figure 4L–O) *n* = 5‐6. Statistical significance was determined using unpaired Student's two‐tailed *t* test. Data represent the mean ± s.e.m. For box‐and‐whiskers plots, the center line represents the median, the bounds of the box represent quartiles and the whiskers represent min to max. Statistically significant differences are shown with asterisks as follows: ^*^ *p* values < 0.05; ^**^ *p* values < 0.01; ns, no significant differences. FMT: Fecal microbiota transplantation.

The expansion of gut fungi characterizes intestinal homeostasis disorders in patients with ALD.^[^ ^27^ ^]^ Analysis of Chao1 and Simpson's index showed similar alpha diversity in the mycobiome between the HKLJ‐treated group and the ethanol‐fed group (Figure 3F). Further beta diversity analysis indicated a shift in the mycobiome after HKLJ supplementation (Figure 3G; Figure S5, Supporting Information). It was found that long‐term ethanol intake changed the gut mycobiome, especially by enriching the alcohol‐tolerant /producing fungus *Pichia kudriavzevii* (Figure 3H–J). Treatment with HKLJ effectively suppressed the alcohol‐induced overgrowth of *P. kudriavzevii* in the gut (Figure 3H–J). To further compare the influences of the gut microbiota from the HKLJ‐treated ALD mice and the ethanol‐fed model mice on alcoholic liver injury, fecal microbiota transplantation (FMT) was performed in NIAAA pseudo germ‐free animals (**Figure** **4**A). The antibiotics‐treated mice receiving faecal microbiota from the HKLJ‐treated ALD mice showed much lower levels of ALT, AST, and hepatic triglyceride, and less hepatic fat deposition, as compared to the antibiotics‐treated mice receiving faecal microbiota from ethanol‐fed model mice (Figure 4B–F).

Significant reduction of butyrate level in the gut was characterized as one of the main features of gut dysbiosis in ALD patients and animals.^[^ ^28^, ^29^ ^]^ In this study, HKLJ supplementation prevented this decrease (Figure 4G; Figure S6, Supporting Information). We cultured two commensals enriched in the HKLJ‐treated group, *F. rodentium*, and *B. pseudolongum*, and assayed their ability to generate SCFAs. As a result, *F. rodentium* was confirmed to be a butyric acid‐producer, and *B. pseudolongum* was identified as an acetic acid‐producing bacterium (Figure 4H). Intestinal butyrate plays important roles in maintaining hypoxia in the lumen by activating the epithelial PPARγ to control the expansion of opportunistic gut pathogens and retaining gut barrier functions.^[^ ^30^, ^31^ ^]^ A higher protein level of nuclear PPARγ was detected in the intestinal epithelia of HKLJ‐treated mice than in those of ethanol‐fed model mice (Figure 4I). Consistent with the changes in PPARγ protein expression, HKLJ supplementation reduced the mRNA levels of intestinal *Nos2* (Figure 4J). In the following assay using the NIAAA model, supplementation with butyrate sodium produced beneficial effects against ALD similar to those of FMT from HKLJ (Figure 4K–O). Butyric acid contributes to the alleviation of ALD injuries by suppressing the growth of opportunistic gut pathogens and maintaining gut barrier integrity. Based on the above analysis, supplementation with HKLJ in ethanol‐fed mice is beneficial to correct ethanol‐induced gut dysbiosis.

### 2.3 Enhanced IL‐22 Production Contributes to the Benefits of Heat‐killed *L. Johnsonii*

**Figure 5:**
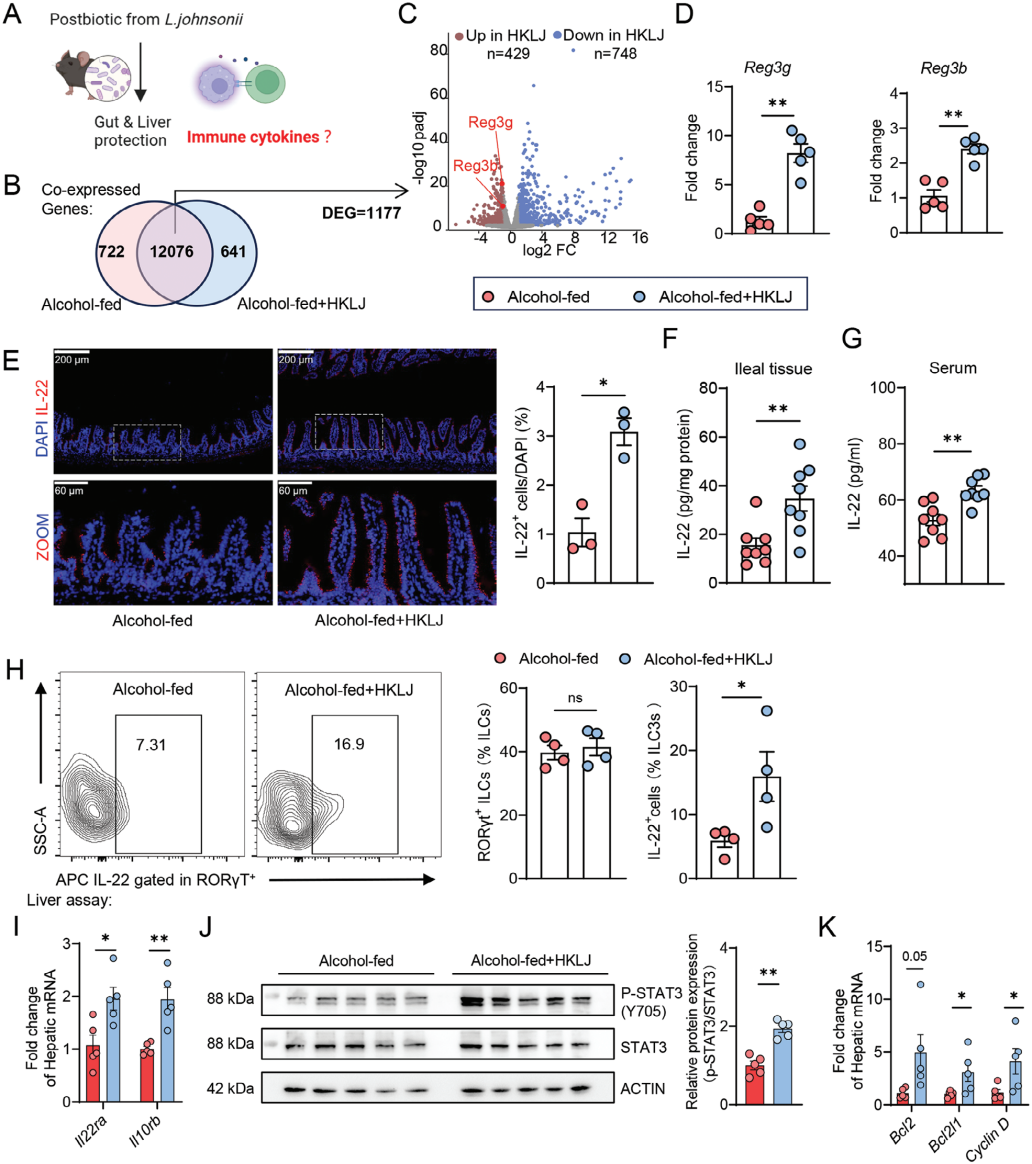
Heat‐killed *L. johnsonii* facilitates intestinal secretion of antimicrobial peptides and hepatocyte repair by promoting IL‐22 production. A) Schematic diagrams of Figure 5 (created by BioRender.com). B) Intestinal co‐expressed genes in ALD mice with administration of vehicle or HKLJ. C) Volcano plots of differentially expressed genes (DEGs) in mouse intestine (blue, downregulated; red, upregulated; change > 2‐fold, *p* < 0.05). D) Ileal mRNA expression levels of *Reg3* *g* and *Reg3b*, normalized to *Gapdh*. E) Representative immunofluorescence staining of IL‐22 and quantitative analysis for IL‐22‐postive cells in ileums. F) Levels of IL‐22 in ileal tissue. G) Levels of IL‐22 in serum. H) Representative flow cytometry plots of IL‐22‐postive ILC3s in the ileal lamina propria. I) Hepatic mRNA expression levels of *Il22ra, Il10rb*, normalized to *Gapdh*. J) Hepatic protein expression of p‐STAT3 and STAT3 in the mice by western blot assay, normalized to β‐actin. K) Hepatic mRNA expression levels of *Bcl2, Bcl2l1*, and *Cyclin D*, normalized to *Gapdh*. Each dot represents data from individual biological replicate. Biological replicate numbers in each group are as follows: (Figure 5B‐5C) *n* = 4, (Figure 5D) *n* = 5, (Figure 5E) *n* = 3, (Figure 5F and 5G) *n* = 8, (Figure 5H) *n* = 4, (Figure 5I‐5K) *n* = 5. Statistical significance was determined using unpaired Student's two‐tailed *t* test, and data represent the mean ± s.e.m., unless otherwise indicated. Statistically significant differences are shown with asterisks as follows: ^*^ *p* values < 0.05; ^**^ *p* values < 0.01; ns, no significant differences. ILC3s: group 3 innate lymphoid cells.

To identify the mechanism by which HKLJ alleviates ALD, we performed RNA‐seq data analysis of intestinal gene expression profiles (**Figure** **5**A). A total of 10276 co‐occurring genes were found between the alcohol‐fed group (*n* = 4) and the HKLJ group (*n* = 4), of which 1177 genes were differentially expressed (Figure 5B). This difference caused changes in the transcriptional profiles (Figure S7A,B). Gene set enrichment analysis (GSEA) revealed the upregulated tight junction‐associated pathways in the HKLJ‐treated group (Figure S7C, Supporting Information), which further confirmed the reinforcement of gut barrier function. Among the differentially expressed genes (DEGs), 429 genes were up‐regulated and 748 genes were down‐regulated after treatment (Figure 5C). In the HKLJ‐upregulated gene set, two antimicrobial peptide genes (*Reg3* *g* and *Reg3b*) were noted to be significantly elevated, respectively (Figure 5D; Figure S7D, Supporting Information). Early studies have illustrated that IL‐22 is a critical factor for regulating the expression of Reg3 family proteins and maintaining gut integrity.^[^ ^32^ ^]^ Here, we measured the expression of intestinal IL‐22 by immunofluorescence, as well as the levels of IL‐22 in the small intestine tissue and circulation by ELISA. Up‐regulated expression and higher levels of IL‐22 were detected in the HKLJ‐treated group (Figure 5E–G). Intestinal IL‐22 is mainly produced by group 3 innate lymphoid cells (ILC3s) in the gut.^[^ ^33^ ^]^ Further flow cytometric analysis of intestinal lamina propria cells showed that the number of IL‐22^+^ ILC3s (gated in RORγT^+^) was markedly increased after HKLJ supplementation (Figure 5H; Figure S8, Supporting Information). In an early ALD study, IL‐22 was reported to alleviate ALD injury via upregulating hepatic signal transducer and activator of transcription (STAT) 3.^[^ ^34^ ^]^ Herein, the gene expression of hepatic IL‐22 receptors (*Il22ra* and *Il10rb*) was up‐regulated 1.8 fold and 1.9 fold, respectively (Figure 5I). Compared to that in ethanol‐fed mice, an activation of the STAT3 pathway in the HKLJ‐treated group was verified by the relatively higher level of phosphorylated STAT3 in the liver (Figure 5J). Correspondingly, the mRNA levels of genes downstream of STAT3 and involved in hepatic cell proliferation and repair were increased (Figure 5K). Overall, HKLJ is capable of upregulating intestinal IL‐22 secretion, thus reinforcing gut barrier function and repairing alcohol‐induced liver injury.

### 2.4 Cell Precipitate from *L. Johnsonii* Alleviate ALD Injuries via Activation of NOD2‐IL‐22 Signaling

**Figure 6:**
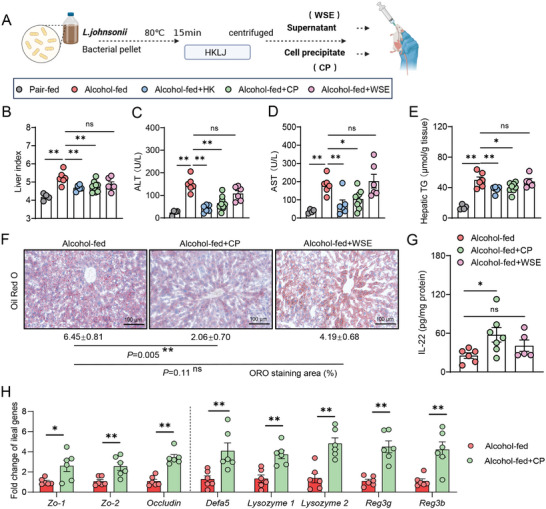
Identification of crude active components in heat‐killed *L. johnsonii*. A) Schematic diagrams of crude extraction about *L. johnsonii* (created by BioRender.com). B) Liver‐to‐body weight ratio at the time of sacrifice. C) Serum levels of ALT. D) Serum levels of AST. E) Triglyceride levels in liver tissue. F) Staining of liver sections for oil red O and quantitative analysis. G) Levels of IL‐22 in ileal tissue. H) Ileal mRNA expression levels of *Zo‐1*, *Zo‐2*, *Occludin*, *Defa5*, *Lysozyme1*, *Lysozyme2*, *Reg3* *g* and *Reg3b*, normalized to *Gapdh*. Each dot represents data from individual biological replicate. Biological replicate numbers in each group are as follows: (Figure 6B–E) *n* = 4‐9, (Figure 6F) *n* = 4‐6, (Figure 6G) 5–7, (Figure 6H) *n* = 6. Statistical significance was determined using one‐way analysis of variance (Figure 6B–G) or unpaired Student's two‐tailed *t* test (Figure 6H), and data represent the mean ± s.e.m., unless otherwise indicated. Statistically significant differences are shown with asterisks as follows: ^*^ *p* values < 0.05; ^**^ *p* values < 0.01; ns, no significant differences. CP: Cell precipitate; WSE: Water‐soluble extracts.

In order to gain insight into the effective components of HKLJ, HKLJ was suspended in a buffer solution and separated into obtain water‐soluble extracts (WSE) and cell precipitate (CP), as shown in the schematic diagram (**Figure** **6**A). In the NIAAA model mice, both HKLJ and CP significantly reduced the liver index, ALT, AST, and hepatic triglycerides, whereas WSE from HKLJ had little effect (Figure 6B–E). Oil red O staining of liver sections also illustrated that CP from HKLJ significantly decreased fat deposition in the livers of ALD (Figure 6F). Consistent with HKLJ, CP from HKLJ promoted IL‐22 secretion, whereas WSE did not influence IL‐22 in the gut (Figure 6G). Meanwhile, CP from HKLJ markedly upregulated the transcriptional levels of tight junction proteins, gut defensins, lysozyme, and antimicrobial peptides (*Reg3* *g* and *Reg3b*), similar to those in HKLJ (Figure 6H).

**Figure 7:**
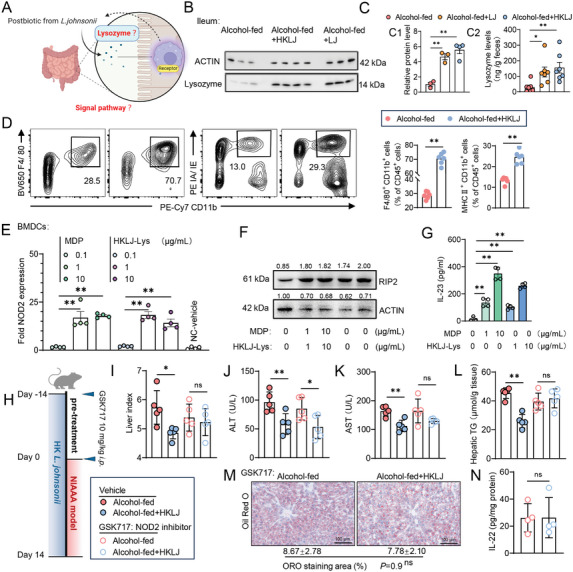
Postbiotics from *L. johnsonii* alleviates ALD via activation of the NOD2‐IL‐22 relays. A) Schematic diagrams of Figure 7 (created by BioRender.com). B) The western blot assay of ileal lysozyme, normalized to β‐actin. C) 1) The protein expression levels of lysozyme in the ileum and 2) levels of lysozyme in the rodent feces. D) Representative flow cytometry plots of F4/80‐postive or IA/IE‐positive (MHC II‐positive) myeloid cells (CD11b‐postive gated) in ileum, and quantification analysis. E) The expression of NOD2 activated by HKLJ‐Lys. Data are presented as fold change relative to vehicle‐treated group (negative control, NC). F) The protein expression of RIP2 in the BMDCs were detected by western blot assay, normalized to β‐actin. G) Levels of IL‐23 in the culture supernatant of BMDCs were detected by ELISA. H) Schematic diagrams of NIAAA mouse model with GSK717 intervention. I) Liver‐to‐body weight ratio at the time of sacrifice. J) Serum levels of ALT. K) Serum levels of AST. L) Triglyceride levels in liver tissue. M) Staining of liver sections for oil red O and quantitative analysis. N) Levels of IL‐22 in ileal tissue. Each dot represents data from individual biological replicate. Biological replicate numbers in each group are as follows: (Figure 7B, C_1_) *n* = 3, (Figure 7C_2_) *n* = 7, (Figure 7D) *n* = 6, (Figure 7E‐7G) *n* = 4, (Figure 7I‐7L) *n* = 5, (Figure 7M and 7N) *n* = 4. Statistical significance was determined using one‐way analysis of variance or unpaired two‐tailed *t* test, and data represent the mean ± s.e.m., unless otherwise indicated. Statistically significant differences are shown with asterisks as follows: ^*^ *p* values < 0.05; ^**^ *p* values < 0.01; ns, no significant differences. BMDCs: Bone marrow derived dendritic cells; MDP: Muramyl dipeptide; NOD2: Nucleotide binding Oligomerization Domain 2; RIP2: Receptor‐interacting protein kinase 2.

In light of the change in the lysozyme expression, we deduced that HKLJ and CP enhance the expression and secretion of lysozyme from the host intestine. Lysozymes, mainly present in the intestinal lumen, facilitate the release of antigenic components from bacteria, thereby eliciting an immune response (**Figure** **7**A). Subsequently, lysozyme levels in ileal tissues and feces of LJ‐ and HKLJ‐treated ALD mice were assayed, revealing that postbiotics administration significantly increased the level of lysozyme in the intestinal mucosa and lumen (Figure 7B–C). At the interface between the mucosa and the microbiome, a high level of lysozyme is secreted from myeloid dendritic cells stimulated by microbial antigens.^[^ ^35^ ^]^ In the present study, HKLJ supplementation significantly elevated the proportions of both F4/80^+^ myeloid cells (macrophages) and MHC II^+^ myeloid cells (dendritic cells, DCs), indicating increases in the activation and antigen presentation of myeloid cells (Figure 7D; Figure S9, Supporting Information). Considering the increased expression of intestinal lysozyme by HKLJ and its CP, we treated the precipitates of HKLJ with lysozyme in vitro to give the corresponding cell lysates (HKLJ‐Lys). Using bone marrow‐derived DCs (BMDCs) as an in vitro model, we assayed the activation of the NOD2/RIP2 pathway and the production of IL‐23 (an upstream cytokine of IL‐22) by HKLJ‐Lys with the known NOD2 ligand MDP as positive control. Treatments at 1 and 10 µg mL^−1^ effectively enhanced both the expression of NOD2 and RIP2 (a NOD2 downstream switch), as well as the production of IL‐23 (Figure 7E–G). In the NIAAA experiments, the anti‐ALD effects of HKLJ, except for ALT reduction, were largely blocked by the NOD2 inhibitor GSK717 (Figure 7H–N). Thus, it was evidenced that supplementation with HKLJ induces the expression of intestinal lysozyme, which in turn enhances the release of immunoregulatory components from HKLJ that bind to the NOD2 receptor to induce myeloid cell activation and antigen presentation, triggering the production of IL‐22 from ILC3. The aforementioned data illustrate the intricate interactions between the postbiotics of *L. johnsonii* and ALD mice. Further studies on the immunoregulatory components of *L. johnsonii* are needed.

## 3 Discussion

The discovery and characterization of new postbiotics not only provide insights into the mechanism of action for probiotics but also advance new therapeutic strategies for diseases. In this study, we identified a gut commensal *L. johnsonii*‐derived postbiotics that protects against gut microbiota dysbiosis, mucosal damage, and hepatic injury induced by ethanol. Mechanistically, we demonstrated that daily supplementation with heat‐killed *L. johnsonii* induced the expression of intestinal lysozymes to increase the production of immunoregulatory substances from gut bacteria that subsequently activated the NOD2‐IL‐23‐IL‐22 innate immune axis.

Lysozymes secreted from gut mucosal cells play important roles in liberating NOD1/2 ligands from microbes.^[^ ^36^, ^37^, ^38^ ^]^ In addition, the activation of the NOD2/RIP2 pathway by intestinal ligands positively mediates the enterocytic lysozyme sorting.^[^ ^39^, ^40^ ^]^ Herein, we found that the lysozyme‐lysates from HKLJ enhanced the expression of NOD2 and RIP2 in vitro. In this context, we introduce a feedback loop involving lysozyme and postbiotics. Specifically, administration of HKLJ postbiotics induces the upregulated expression of intestinal lysozymes, which in turn increases the production of immunoregulatory ligands from HKLJ. The increased ligands further trigger NOD2‐related signaling in dendritic cells to promote cellular lysozyme sorting and downstream immune cytokines production. This loop stimulated by HKLJ plays a crucial role in mediating its anti‐ALD effects. In addition to the NOD2 pathway, *L. johnsonii* was reported to activate TLR1/2 in macrophages for relieving colitis,^[^ ^20^ ^]^ suggesting that other immune signaling pathways be involved in the interaction between this symbiont and host. The immunoregulatory components originated from *L. johnsonii* and their regulatory pathways beyond NOD2 deserve further investigation.

Interleukin‐22 (IL‐22) plays important roles in regulating tissue regeneration and repair, pathogen clearance, and immune homeostasis. We found that heat‐killed *L. johnsonii* supplementation enhanced the level of IL‐22 in ethanol‐fed mice. In the field of ALD treatment, IL‐22 agonists have been demonstrated to be effective in the clinic.^[^ ^41^ ^]^ We also demonstrated that the lysozyme‐lysates of *L. johnsonii* stimulated IL‐23 production from the activating DCs, which is the upstream cytokine of IL‐22. DCs and ILC3s hook up through IL‐23 to maintain the production of intestinal IL‐22 in heat‐killed *L. johnsonii*‐treated ethanol‐fed mice, further upregulating the expression of gut tight junction proteins and defense proteins and promoting hepatocyte regeneration via activation of STAT3.

Long‐term alcohol consumption greatly disturbs the gut microbiota, leading to the overexpansion of potential pathogens and a reduction in butyrate‐producing bacteria.^[^ ^28^, ^29^ ^]^ Moreover, the ethanol‐induced gut microbiota is confirmed to be a disease‐driving factor exacerbating experimental ALD symptoms by FMT experiment.^[^ ^42^ ^]^ Owing to a heat‐killed *L. johnsonii*‐induced increase in the expression of Reg3 family defense proteins, the ethanol‐caused expansion of enteric *Escherichia coli* and *Helicobacter* sp. was effectively suppressed. These pathogens reportedly boost the progression of ALD.^[^ ^16^, ^43^, ^44^ ^]^ Interestingly, heat‐killed *L. johnsonii* increased the abundance of SCFA‐producing *Faecalibaculum rodentium* and *Bifidobacterium pseudolongum* in the gut of ethanol‐fed mice. In an early study, the SCFA‐producing *Faecalibaculum spp*. and *Bifidobacterium spp*. were suggested to be cross‐feeding.^[^ ^45^ ^]^ The *F. rodentium* and *B. pseudolongum* strains have been demonstrated to be beneficial for treating gastrointestinal and hepatic disorders.^[^ ^46^, ^47^ ^]^ In addition to the expansion of facultative bacteria, the overgrowth of gut fungi was confirmed to accelerate the progression of ALD via enhancing pro‐inflammatory signaling through the secreted toxins or β‐glucan.^[^ ^3^, ^27^, ^48^ ^]^ In human cohorts, the abundance of *Pichia* sp. was found to be elevated in alcoholic hepatitis and alcoholic cirrhosis patients.^[^ ^27^ ^]^ Herein, supplementation of heat‐killed *L. johnsonii* significantly restricted the ethanol feeding‐induced overgrowth of *Pichia kudriavzevii* (early named *Candida krusei*). It has been demonstrated that gut microbial butyrate triggers PPARγ signaling on the gut epithelium to maintain an intestinal hypoxic environment and control gut microbiota homeostasis,^[^ ^30^ ^]^ which assists in the amelioration of ALD. Consistent with the observed changes in the gut microbiome, we found that heat‐killed *L. johnsonii* increased the level of gut butyrate and activated the epithelial PPARγ signaling pathway. The maintenance of gut hypoxia helps to control the expansion of facultative bacterial pathogens and fungi in the gut of ALD mice.

In summary, postbiotics derived from gut commensal *L. johnsonii* protect against ethanol‐disrupted gut barrier functions, restore ethanol‐induced gut microbiota dysbiosis, and attenuate hepatic injury by triggering the gut NOD2‐mediated innate immune pathway in ALD mice. Increased IL‐22 was confirmed to be an effector mediating the aforementioned benefits of *L. johnsonii* postbiotics. These anti‐ALD phenotypes conferred by *L. johnsonii* make this symbiont a promising candidate for clinical interventions. However, being a study in the mouse model, a randomized controlled trial with a placebo is needed to assess the safety and efficacy of HKLJ postbiotics in a clinical setting. Altogether, this study in combination with earlier research on probiotics, postbiotics, and prebiotics, supports the development of microbiome‐based therapeutics for the treatment of ALD.

## 4 Experimental Section

### Animal Models and Animal Ethics

The research complied with all relevant ethical regulations. The animal experiments were approved by the research ethics committee of the Institute of Microbiology, Chinese Academy of Sciences (permit APIMCAS2023115). Age‐matched male C57BL/6JGpt mice (strain no. N000013; aged 8–10 weeks; body weight of 22–25 g) were purchased from GemPharmatech Co., Ltd. (Nanjing, China). Two mice were housed in an independent animal facility under specific‐pathogen‐free conditions and subjected to the chronic feeding model or chronic plus‐binge model (NIAAA model).

For the chronic feeding model,^[^ ^49^ ^]^ mice were fed with a Lieber‐DeCarli liquid diets (BioServ, NO. F1258SP, USA) containing ethanol (alcohol‐fed) or isocaloric control liquid diets containing maltodextrin (Aladdin, NO. M305460, China) (pair‐fed) for 8 weeks. In detail, mice were fed the Lieber‐DeCarli liquid diets by gradually increasing the ethanol concentration to 4.5% (wt/vol) ethanol (24% calories in the diet) in the first two weeks and then gradually increasing the ethanol concentration to 5.6% (wt/vol) ethanol (30% calories in the diet) in the last two weeks.

For the chronic plus‐binge alcohol feeding model (NIAAA model),^[^ ^49^ ^]^ 8‐week‐old mice were fed the Lieber‐DeCarli liquid diets. The content of alcohol in the Lieber‐DeCarli liquid diets gradually increased from 0% (wt/vol) to 5% (wt/vol) over 0–5 days and remained at 5% for the subsequent 10 days. On the last day, the mice were gavaged with a single dose (5 g per kilogram of body weight) of ethanol at 9:00 a.m. and euthanized 9 h later. Pair‐fed control mice were fed an iso‐caloric liquid diet and were euthanized 9 h after intragastric administration of the iso‐caloric maltodextrin solution.

### Microbe Strains

*Lactobacillus johnsonii* (LJ) (CGMCC No. 27014) which was previously isolated from feces of a healthy human infant was used for this study (APIMCAS2017049). The bacterial strains were plated on De Man, Rogosa, and Sharpe (MRS) agar medium plates (HB0384‐5, Hopebio, China) and incubated in an anaerobic atmosphere (15% H_2_, 5% CO_2_, and 80% N_2_) for 48 h at 37 °C. Prior to use, mono‐clones were picked and inoculated with fresh sterile MRS broth. The bacterium was anaerobically incubated for 24 h at 37 °C to the logarithmic growth phase (Log‐phase).

*Faecalibaculum rodentium* and *Bifidobacterium pseudolongum* were also isolated from the ileal mucosa of C57BL/6J mice and preserved in the laboratory. These strains were anaerobically incubated in modified Gifu Anaerobic Medium (mGAM) (HB8518, Hopebio, China) for 48 h at 37 °C. Prior to use, mono‐clones were picked and inoculated with fresh sterile mGAM broth, anaerobically incubated for 24 h at 37 °C to the Log‐phase.

### Extraction of *L. Johnsonii* Cell Components

The *L. johnsonii* was grown as a 50 mL culture in MRS broth to mid‐logarithmic phase (1 × 10^8^ CFU mL^−1^) and then pelleted by centrifugation at 5000 rpm for 20 min at 4 °C. Cell pellets were then rewashed with 50 mL of phosphate buffer saline (PBS), resuspended in 5 mL of PBS, and stored at 4 °C until use. The bacterial pellet of *L. johnsonii* was then resuspended in phosphate buffer saline (PBS) and pasteurized at 85 °C for 15 min. Further centrifugation at 13 000 rpm for 10 min provided the corresponding supernatant and the cell precipitate.

To get the cell components, the cell precipitate of *L. johnsonii* was resuspended in 1 mL of 5% SDS in PBS and then boiled for 25 min. After centrifugation at 20 000 rpm, the pellets were washed with MilliQ water to remove SDS traces. Next, the pellets were sequentially enzymatically treated with 2 mg mL^−1^ of proteinase K (P109033, Aladdin, China) in PBS for 30 min at 37 °C; 200 µg mL^−1^ of trypsin (T105532, Aladdin, China) in PBS for 2 h at 37 °C with shaking; 10 µg mL^−1^ of bi‐nuclease (B401536, Aladdin, China) in PBS for 1 h at 37 °C. After boiling for 15 min, the suspension was centrifugated at 20 000 rpm for 10 min. The insoluble fractions were digested with lysozyme (A610308, BBI, China) for 18 h at 37 °C with shaking. The resulting lysates (HKLJ‐Lys) were boiled for 15 min and lyophilized.

### Histopathological Analysis

Fresh liver left lobe tissue was resected and fixed with paraformaldehyde (4%, pH 7.4). A portion of the tissue was first embedded in paraffin and then cut into 4 µm sections, followed by hematoxylin and eosin (H&E) staining. The remaining part was wrapped in optimal cutting temperature (OCT) compound and cut into 10 µm sections, followed by oil red O and Sirius red staining (Solarbio, G1261 and G1472, China). Quantitative analysis of liver histologic scores was performed according to a combination of the following two standards: degree of inflammation in the confluent area: 0, normal; 1, mild inflammatory infiltrate; 3, moderate inflammatory infiltrate. Steatosis score: 0, normal; 1, a small amount of ballooning degeneration; 3, degeneration involving more than 25% of the liver's left lobe. The fresh ileum was also sectioned and stained following the standard histological procedure described above. All sections were observed by a Zeiss Imager A2‐M2 microscope (Carl Zeiss AG, Germany). At least 3 fields of view per section of each intestine were measured and the images were manually delineated and analyzed using Image‐Pro Plus 6.0 (from the NIH, USA).

### RNA Extraction and RT‐qPCR

Total RNA was extracted from the ileal or liver tissues of animals using TRIeasy Total RNA Extraction Reagent (Yeasen, 10606ES60, China) according to the manufacturer's instructions. cDNA was reverse transcribed from 500 ng of total RNA using Hifair II 1st Strand cDNA Synthesis SuperMix (Yeasen, 11123ES60, China). The target primers used are listed in Table S1 (Supporting Information). RT‐qPCR was performed using NovoStar SYBR qPCR SuperMix Plus (Novoprotein, E096‐01A, China), and amplification was initiated in a CFX Connect Real‐Time PCR Detection System (Bio‐Rad, 1855201, USA). For each target gene, the housekeeping gene glyceraldehyde‐3‐phosphate dehydrogenase (*gapdh*) was used for internal normalization.

### Enzyme‐Linked Immunosorbent Assay (ELISA)

ELISA kits were used to detect the protein levels of cytokines (TNF‐a, IL‐6, IL‐1β, IL‐10, and IL‐22), lysozyme, and lipopolysaccharide (LPS) in the rodent serum or feces, as well as the level of IL‐23 in the cell culture supernatants, separately. Whole blood was collected from mice using the inner canthus method. Serum was prepared by centrifugation at 3000 rpm for 15 min at room temperature. The 100 mg of feces was collected and homogenized (65 Hz) with 1 mL PBS using zirconia beads. The supernatant was separated from the homogenized suspension by centrifugation at 13 000 rpm for 15 min and placed at 4 °C. Cell culture supernatants were collected by centrifugation at 3000 rpm for 5 min. The experiment was carried out according to the manufacturer's instructions.

### Western Blot Analysis

These protein lysates were separated by sodium dodecyl sulphate polyacrylamide gel electrophoresis (SDS‐PAGE) and then transferred to polyvinylidene difluoride (PVDF) membranes. After being blocked by 5% skimmed milk (Biofroxx, 1172GR500, Germany), this membrane was incubated with primary antibody at 4 °C overnight. After incubation with a corresponding second antibody, the whole membrane was immersed in a super sensitive ECL luminescence reagent (Meilunbio, MA0186, China). The protein bands in the membrane were detected and quantified using a fully automated chemiluminescence image analysis system (Tanon, 5200, China) and Image J software.

### Cecal DNA Extraction and Sequencing

Cecal tissue was obtained at −80 °C, and the cecal contents were transferred to sterile 1.5 mL centrifuge tubes. The bacterial genomic DNA of the contents was isolated using the Stool Genomic DNA Kit (CWBIO, CW2092, China). Genomic DNA purity was detected by a Nano‐Photometer spectrophotometer (IMPLEN, USA). The V3‐V4 region of the bacterial 16S rRNA genes was amplified by polymerase chain reaction (PCR) using Platinum Hot Start PCR master mix (Invitrogen, 13000012, USA), primers, and genomic DNA (20 ng). After the individual quantification step, the amplicon libraries were pooled in equal amounts, and paired‐end 2–250 bp sequencing was performed using the Illumina NovaSeq‐PE250 platform at Personal Biotechnology Co., Ltd (Shanghai, China). A similar approach was applied to the mycobiome targeting the internal transcribed spacer (ITS) V1 region of fungal ribosomal genes, as well as an optimized and standardized protocol for the preparation of ITS1 sample libraries.

Alpha diversities were analyzed using the Chao index and the Simpson index calculated with observed amplicon sequence variants (ASVs). Beta diversity was visualized via principal coordinate analysis (PCoA), and assessed using unweighted UniFrac distance or Jaccard distance. Taxonomic analysis was performed using the QIIME 2 feature‐classifier trained on the NCBI database. For precise classification, each unique ASV was searched against the NCBI database by the BLAST tool to find the closest class group at the species level according to percent identity. Linear discriminant analysis effect size (LEfSe) was performed with the default parameters to detect diverse taxa between groups.

### Quantification of Short‐Chain Fatty Acids (SCFAs)

Individual mice were placed in an empty sterile facility (without bedding) and removed after 45 min to collect fecal pellets, which were then stored at –80 °C for gas chromatography‐mass spectrometry (GC‐MS) analysis. For the extraction of SCFAs from feces, 50 mg freeze‐dried fecal samples were homogenized in 1 mL of methanol (Sigma, CAS No. 67‐56‐1, USA) on ice for 10 min. The supernatant enriched with SCFAs was isolated by centrifugation. For the extraction of SCFAs from the medium, bacterial broth cultured to the log phase was treated with an equal amount of ethyl acetate (Sigma, CAS No. 141‐78‐6, USA). After shaking, the organic phase of the supernatant was obtained by centrifugation. The extractions were aliquoted into 2 mL ampoules after passing through 0.2 µm nylon filter membranes (Agilent, 5191‐4341, USA) for GC‐MS analysis (Agilent, 7890A/5975C, USA). GC‐MS analysis was performed according to a previously described method.^[^ ^50^ ^]^ GAM broth was used as the negative control.

### Isolation of Intestinal Lamina Propria Cells

The protocol for isolating intestinal lamina propria cells has been slightly modified from the previous description.^[^ ^51^ ^]^ Collected intestinal pieces after removing Peyer's patches were shaken (150 g) for 10 min at 37 °C in Hank's buffer (Gibco, 14175095, USA) containing 5 mM EDTA (Invitrogen, AM9262, USA), 1 mM DTT (Solarbio, D8220, China) to deplete the epithelial layer and mucus. Mucus‐depleted intestinal pieces were digested in RPMI‐1640 medium (Gibco, C11875500BT, USA) with 5% v/v FBS (Meilun‐bio, PWL001, China), 1.0 mg mL^−1^ collagenase II and III (Worthington, WBC‐LS004176 and WBC‐LS004182, USA), and 0.2 g L^−1^ DNase I (Roche, 10104159001, USA) for 30 min at 37 °C. After centrifugation, the intestinal lamina propria cells were passed through a 70 µm cell filter and washed twice with Hank's buffer for flow cytometry assay.

### Flow Cytometry

After 6 h of incubation with RPMI‐1640 medium containing cell activation cocktail (Biolegend, 423304, USA), intestinal lamina propria cells were blocked with anti‐CD16/32 (BD, 553141, USA) antibody for 5 min, and then incubated on ice with Zombie NIR fixable viability kit (Biolegend, 423106, USA) for 15 min under dark. During the incubation, the cells surface was stained with the antibody mixture for 30 min at 4 °C. Finally, cells were permeabilized and fixed with transcription factor buffer (BD, 562574, USA) and TF perm/wash buffer (BD, 9008102, USA) before staining with the antibody mixture (omitted in DCs protocol). Finally, the stained cells were resuspended in Stain buffer (BD, 554656, USA) and were detected in a Flow Cytometer (BD, LSR‐Fortessa, USA). The analysis was completed using FlowJo software (Tree Star Inc., USA).

### Cell Culture

C57BL/6J mice were submerged in 75% alcohol for 5 min after euthanasia (CO_2_ asphyxiation). In a sterile hood, the cavities of the tibia and femur were cracked open, and the bone marrow was flushed out with PBS and blown into a single‐cell suspension. The collected cells were cultured in RPMI‐1640 complete medium containing 20 ng mL^−1^ of granulocyte‐macrophage colony stimulating factor (GM‐CSF) (MCE, HY‐P7361, USA) and 1 ng mL^−1^ of IL‐4 (Novoprotein, P07750, China).^[^ ^52^ ^]^ The cells were cultured at 37 °C in a humidified environment with 5% CO_2_, and were induced into Bone marrow‐derived dendritic cells (BMDCs) after one week. BMDCs were collected after 12 h of drug stimulation and stored at −20 °C for assay.

### Statistics

All statistical analyses were conducted using the R package or GraphPad Prism software (version 8.0.2). The data were presented as the mean ± SEM or as individual data points unless otherwise specified. The normality of the probability distribution was confirmed by the Shapiro Wilk test and the variance in the probability distribution was confirmed by the Brown‐Forsythe test to determine the appropriate statistical analysis method using SPSS (version R27.0.1.0). Statistical comparisons between only two groups were conducted using unpaired Student's two‐tailed *t*‐test (normal probability distribution and homogeneity of variance) or the Mann Whiney U test (abnormal probability distribution or heterogeneity of variance). For comparisons of more than two groups, a one‐way analysis of variance (ANOVA) test (normal probability distribution) or non‐parametric Kruskal Wallis test (abnormal distribution) followed by a post hoc Dunnett's multiple comparison test using Alcohol‐fed group data as control was performed. Statistically significant differences are shown with asterisks as follows: ^*^ *p* values < 0.05; ^**^ *p* values < 0.01; ^ns^ *p* values > 0.05.

Additional detailed methods are provided in the Supporting Information.

## Conflict of Interest

The authors declare no conflict of interest.

## Author Contributions

R.Y. performed most of the experiments, analyzed and interpreted data, and prepared the submitted manuscript. T.W. performed some animal experiments. J.S. isolated and identified all strains. H.D. contributed to the critical discussion of the project. Y.Z. provided technical support for flow cytometry and cell assay. N.L. provided the design of animal models and contributed to manuscript revision. H.L. conceived, designed, and supervised the study and revised the manuscript.

## Supporting information



Supporting Information

## Data Availability

The data that support the findings of this study are available in the Supporting Information of this article.
