# Supplementary material for: Postbiotics From Lactobacillus Johnsonii Activates Gut Innate Immunity to Mitigate Alcohol‐Associated Liver Disease
Source: Adv Sci (Weinh). 2024 Nov 22;12(2):2405781. doi: 10.1002/advs.202405781 (PMC11727117; doi:10.1002/advs.202405781)
Supplement: Supplementary file 1 — Supporting Information [file ADVS-12-2405781-s001.docx]

**Supporting Information for:**

**Postbiotics from *Lactobacillus johnsonii*** **Activates Gut** **Innate Immunity to Mitigate** **Alcohol-associated Liver Disease**

Ruopeng Yin, Tao Wang, Jingzu Sun, Huanqin Dai, Yuting Zhang, Ningning Liu, Hongwei Liu*

**This** **file includes:**

Method details

Figure S1 to S9

References

**Method details**

**Biochemical analyses**

The levels of alanine aminotransferase (ALT) and aspartate aminotransferase (AST) in the serum were measured using commercial kits. Liver tissues were weighed and homogenized in PBS. After centrifugation (11,000 rpm for 30 min, 4°C), the supernatants were collected and used to determine the levels of reduced glutathione (GSH), oxidized glutathione (GSSG), and total triglycerides (TG) according to the instructions of commercially available kits.

**Scanning electron microscope (SEM)**

The bacterium-fixed slides were prepared for scanning according to the previously protocol.^[1]^ The morphology of bacteria was observed and imaged under a SEM system (Hitachi, Regulus 8100, Japan).

**Animal experimental design**

**Animal experiment 1**: Mouse was orally gavaged bacterial suspension containing 2 × 10^8^ colony-forming units (CFUs) of live *L. johnsonii* (LJ) or heat-killed *L. johnsonii* (HKLJ) daily until the end of the chronic feeding model. The control group received PBS.

**Animal experiment 2**: Mouse was orally gavaged heat-killed *L. johnsonii* (HKLJ) daily until the end of the chronic plus-binge alcohol feeding model. The control group (model group) received PBS.

**Animal experiment 3**: Mice in treatment groups were orally administered with a suspension containing HKLJ, the corresponding water-soluble extracts (WSE), and cell precipitates (CP) from HKLJ every day until the end of the chronic plus-binge alcohol feeding model, respectively. The other groups received PBS as a vehicle.

**Animal experiment 4**: Eight-week-old mice were randomly grouped and given drinking water containing a mixture of antibiotics (1 mg/mL of ampicillin, 0.5 mg/mL of vancomycin hydrochloride, 0.5 mg/mL of neomycin sulfate, 1 mg/mL of metronidazole, 0.05 mg/mL of amphotericin B, and 0.5 mg/mL of sucralose; from Macklin, A830931, V820413, N814740, M813526, A6061, S817766, China) for 7 d. The obtained pseudo germ-free mice were then grouped for the fecal microbiota transplantation (FMT) assay. Mice were gavaged separately with fecal microbial suspension from a chronic plus-binge alcohol feeding model (6-7 mice per group) once a day. Briefly, 100 mg of fecal samples were homogenized in 500 μL of anaerobic PBS and passed through a cell sieve (pore size: 100 μm) to remove undigested-residue. Finally, the resulting suspension was administered to each recipient pseudo germ-free mouse by gavage of 200 μL. The whole transplantation process lasted for two weeks until the end of the chronic plus-binge alcohol feeding model.

**Animal experiment 5**: In experiment of butyrate supplement, 200 mg/kg of sodium butyrate (Macklin, No. S817488, CAS.156-54-7, China) or the same volume of PBS were orally gavaged into mice once daily for 16 days until the end of the chronic plus-binge alcohol feeding model.

**Animal experiment 6**: In the NOD2 inhibitor experiments, 10 mg/kg of the inhibitor GSK717 (Med-Chem-Express, No. HY-136555, CAS. 1595278-21-9, USA) or the same volume of auxiliary vehicle (10% DMSO, 40% PEG300 and 50% PBS) was administered to mouse, respectively, with reference to previous studies.^[2]^ As in animal experiment 2, HKLJ was administered to these mice by gavage daily.

**Immunofluorescence analysis**

For immunohistochemical staining, paraffin sections of animal tissue were deparaffinized with xylene, dehydrated in gradient alcohol, rinsed in distilled water, and retrieved antigen in boiling citrate buffer. The ready sections were blocked with 5% donkey serum for 1 h, followed immediately by sequential incubation with the antibody. Finally, sections were washed and incubated with DAPI (Invitrogen, D1306, USA) and filled with Diamond anti-quenching mix (Invitrogen, P36961, USA). Images were captured using an Eclipse Ni-E/Ni-U microscope (Nikon, Japan) and analyzed with Image-Pro Plus 6.0 (from NIH, USA).

**Extraction of tissue and cell protein**

For extraction of protein from liver, a portion of the left liver lobe was cut, thrown into RIPA lysis buffer (Thermo Scientific, 89900, USA) with phosphatase inhibitor cocktail (Proteintech Group, Inc, PR20015, USA), homogenized as previously described, and quantified using the Quick Start™ Bradford kit (Bio-Rad, 5000202, USA). For the extraction of intestinal epithelial proteins, protocols were carried out with some modifications as previously described^[3]^. Briefly, a 1cm ileum was freed from the animal, longitudinally cut and rinsed in pre-cooled PBS to remove feces and adipose tissue. The intestinal pieces were then transferred into PBS supplemented with EDTA (2.5 mM; Invitrogen, AM9262, USA), followed by incubation at 37 °C under slow rotation for 20 min. The suspensions were filtered, and epithelial cells were collected by centrifugation (1,100 rpm) and used for further analyses. Intestinal epithelial cell samples were extracted with commercial nuclear protein extraction kit (Beyotime, P0027, China). For extraction of protein from BMDCs, 200 μL of RIPA lysis buffer was added to the plate with BMDCs, incubated on ice for 10 min, and cell lysates were obtained by centrifugation of cell suspensions at 4 degrees at 13,000 rpm for 10 min after blowing with a pipette.

**RNA sequencing**

The total RNA of whole ileum tissue was extracted according to the instructions. Total RNA was quantified and assessed for purity using a NanoDrop 2000 Spectrophotometer (Thermo Scientific). The RNA samples were sequenced using the Illumina PE150 platform and gene expression levels were expressed as RPKM (reads per kilobase per million) by Novo-gene. The R package “ggplot2” was invoked to map volcano plot and heatmap about all differentially expressed genes. The dataset was analyzed using the Kyoto Encyclopedia of Genes and Genomes (KEGG) database using a threshold (*P* > 0.05) to identify up and down regulated signaling pathways.

**Detect the expression of NOD2**

The effect of different samples (including lysates and commercial muramyl dipeptide in vitro as described above) on the expression of NOD2 was monitored using BMDCs. Prior to the addition of lysates, induced mature DCs were inoculated in 96-well plates (10,000 cells/well) and incubated at 37°C for 24 hours. Subsequently, lysates were added to make a total volume of 200 μL. After incubation for 12 h, the cells were collected, and NOD2 expression in the cells was detected using RT-qPCR.

**Antibody sources**

For flow cytometry:

anti-mouse CD45-BV650 (Biolegend, 103151, USA)

anti-mouse CD3-FITC (Biolegend, 100203, USA)

anti-mouse NK-1.1-PE (Biolegend, 156503, USA)

anti-mouse CD127-BV711 (Biolegend, 135035, USA)

anti-mouse/human CD11b-PE/Cyanine7 (Biolegend, 101216, USA)

anti-mouse F4/80-BV605 (Biolegend, 123133, USA)

anti-mouse I-A/I-E (MHC Ⅱ)-PE (Biolegend, 107607, USA)

anti-mouse RORγt-BV650 (BD, 564722, USA)

anti-mouse IL-22-APC (Biolegend, 516409, USA)

anti-mouse Gr-1+CD11b+CD45R/B220 (Lineage cocktail)-PE-cy7 (Biolegend, 108415; 101215; 103221, USA)

For immunohistochemistry:

anti-F4/80 monoclonal antibody (Abcam, ab6640, USA, 1:200 dilution)

anti-IL-22 polyclonal antibody (Service-bio, GB11259-100, China, 1:500 dilution)

Cy5 conjugated goat anti-rat antibody (Sigma-Aldrich, AP183C, USA)

Cy5 conjugated goat anti-rabbit antibody (Service-bio, GB21303, China)

For immunoblot:

STAT3 Rabbit pAb (Zen-Bio, No. 251611, dilution 1:1000, China)

P-STAT3 (Tyr705) Rabbit pAb (Zen-Bio, No. 381552, dilution 1:1000, China)

PPAR gamma Rabbit pAb (Zen-Bio, No. 340844, dilution 1:1500, China)

RIP2 Polyclonal antibody (Proteintech, No.15366-1-AP, dilution 1:1000, USA)

PCNA mouse monoclonal-antibody (Easy-bio, BE0029, dilution 1:2000, China)

Lysozyme rabbit monoclonal antibody (AB-clonal, A0641, dilution 1:2000, China)

HRP-conjugated beta-actin antibody (Service-bio, GB15001, dilution 1:3000, China)

HRP-conjugated Goat anti-Rabbit/Mouse IgG(H+L) (Earth Ox, E030120/E030110, dilution 1:10000, USA)

**Commercia kits**

Mouse LPS ELISA kit (ml-Bio, ml037221, China)

Mouse IL-1 beta ELISA Kit (AB-clonal, RK00006, China)

Mouse TNF-alpha ELISA Kit (Proteintech Group, Inc, KE10002, USA)

Mouse IL-10 ELISA Kit (Proteintech Group, Inc, KE10103, USA)

Mouse IL-6 ELISA Kit (Multi-Sciences, EK206, China)

Mouse IL-22 ELISA Kit (Multi-Sciences, EK222, China)

Mouse Lysozyme ELISA Kit (CUSABIO, E11329m, China)

Alanine aminotransferase (ALT) Assay Kit (Jiancheng, C009-2-1, China)

Aspartate aminotransferase (AST) Assay Kit (Jiancheng, C010-2-1, China)

Triglyceride assay kit (Jiancheng, A110-1-1, China)

Reduced glutathione (GSH) Content Assay Kit (Solarbio, BC1175, China)

Oxidized glutathione (GSSG) Content Assay Kit (Solarbio, BC1185, China)

**Supplementary figures**

**
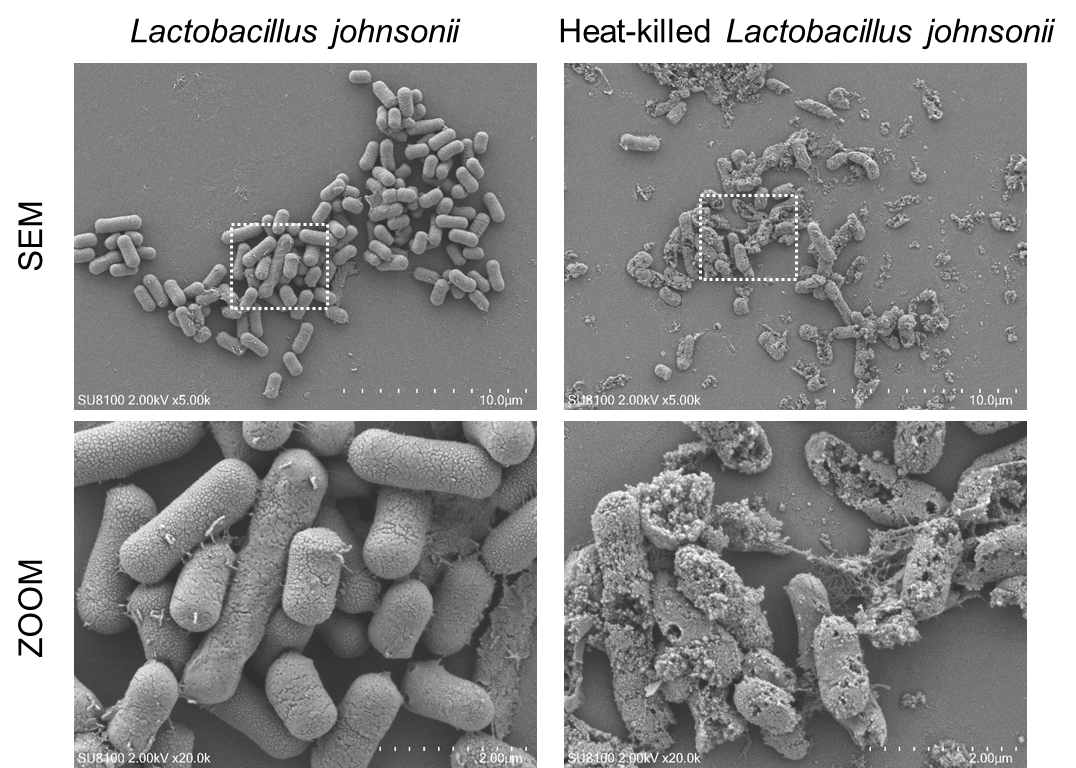
**

**Figure S1**: **Representative SEM images of live *L. johnsonii* and heat-killed *L. johnsonii*.**

**
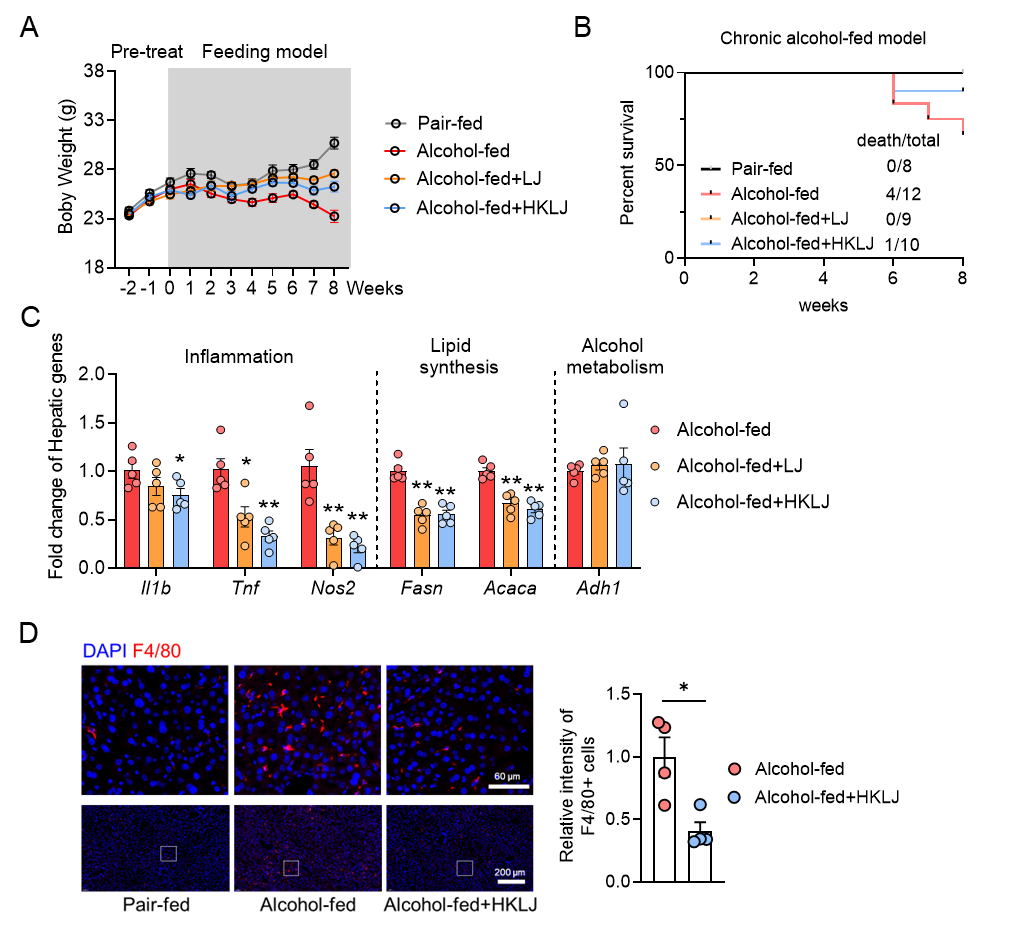
Figure S2**: ***L. johnsonii* relieve ethanol-mediated animal phenotype and liver inflammation.**

(A) Changes in body weight of chronic ALD mice. (B) The survival rates of chronic ALD mice were determined using Kaplan-Meier curves. (C) Hepatic mRNA expression levels of *Il1b*, *Tnf*, *Nos2*, *Fasn*, *Acaca*, and *Adh1*, normalized to *Gapdh*. (D) Representative immunofluorescence staining of F4/80 in liver tissues sections, cell nucleus (DAPI, blue), F4/80 (Cy5, Red). Each dot represents data from individual biological replicate. Biological replicate numbers in each group are as follows: (Figure S2A-S2B) n=8-12, (Figure S2C) n=5, (Figure S2D) n=4. Statistical significance was determined using unpaired one-way analysis of variance, and data represent the mean ± s.e.m., unless otherwise indicated.

**
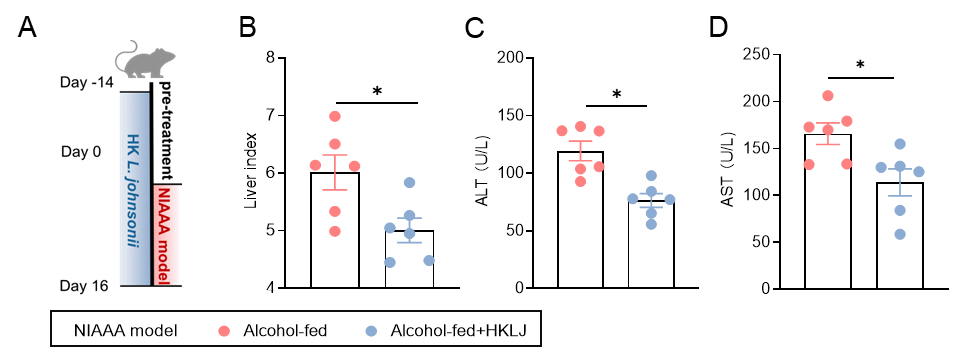
**

**Figure S3**: **Heat-killed *L. johnsonii* (HKLJ) shows remission of liver injury in NIAAA model.**

(A) Schematic diagrams of NIAAA mouse model. (B) Liver-to-body weight ratio at the time of sacrifice. (C) Serum levels of ALT. (D) Serum levels of AST. Each dot represents data from individual biological replicate. Biological replicate numbers in each group are as follows: (Figure S3B-S3D) n=6. Statistical significance was determined using unpaired Student's two-tailed *t* test, and data represent the mean ± s.e.m., unless otherwise indicated.

**
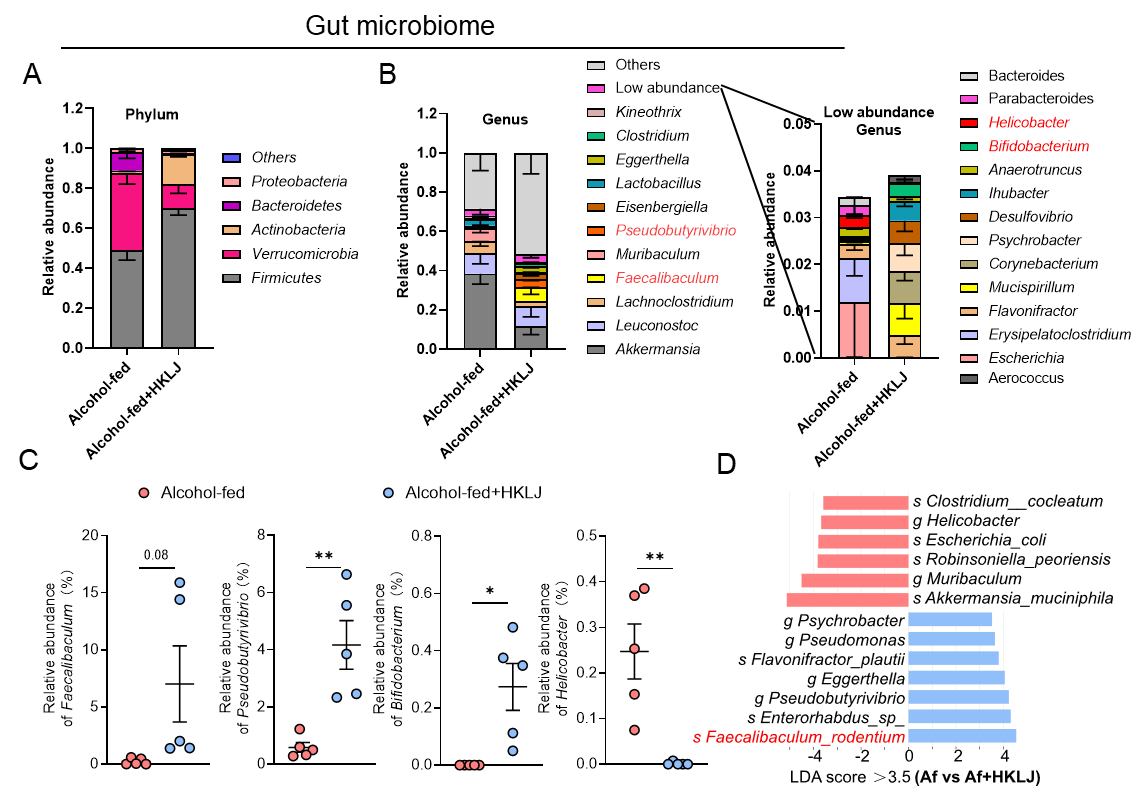
**

**Figure S4**: **HKLJ altered the composition of gut bacteria.**

(A) Taxonomic composition of gut bacteria at the phylum level, as determined with 16S rRNA sequencing. (B) Taxonomic composition of gut bacteria at the genus level, as determined with 16S rRNA sequencing. (C) The relative abundance of *Faecalibaculum*, *Pseudobutyrivibrio*, *Bifidobacterium*, and *Helicobacter* in the gut microbiome. (D) LDA score representing the enriched taxon in the gut microbiome between two group. Only LDA scores (>3.5) are shown. Red indicates enriched taxa in the alcohol-fed group; blue indicates enriched taxa in the HKLJ group. Each dot represents data from individual biological replicate. Five biological replicate numbers in each group. For (C), Statistical significance was determined using unpaired Student's two-tailed t test, and data represent the mean ± s.e.m., unless otherwise indicated.

**
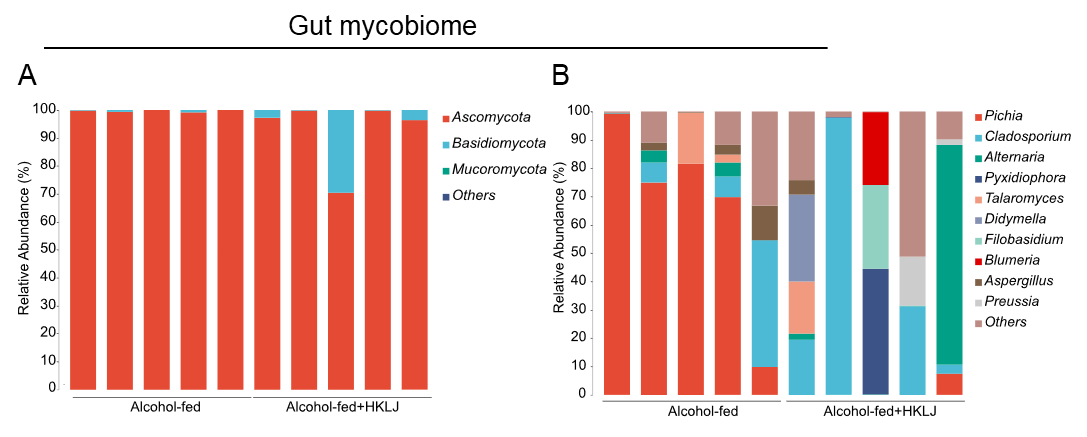
 Figure S5: HKLJ altered the composition of gut fungi.**

(A) Taxonomic composition for composition of gut fungi at the phylum level, as determined with ITS sequencing. (B) Taxonomic composition of gut fungi at the genus level, as determined with ITS sequencing. Five biological replicate numbers in each group.

**Figure S6**: **The content of SCFA metabolites in mouse fecal samples (4 group) was determined by GC-MS.**

Each dot represents data from individual biological replicate. Five biological replicate numbers in each group. Statistical significance was determined using one-Way ANOVA test followed by Dunnett’s multiple comparisons test, and data represent the mean ± s.e.m., unless otherwise indicated.

**
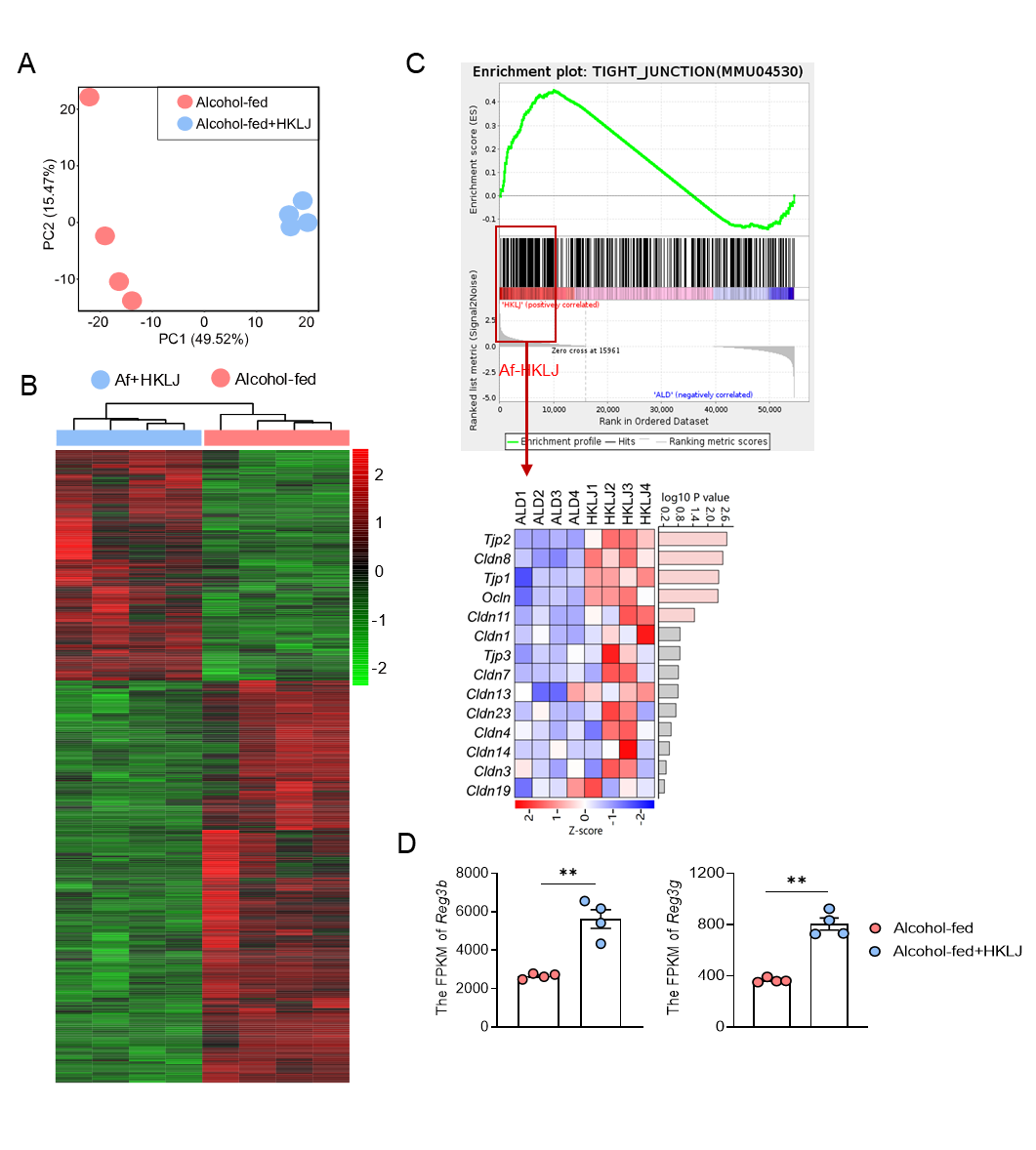
**

**Figure S7**: **Heat-killed *L. johnsonii* *(*HKLJ) alters intestinal transcription profile in ALD mice.**

(A) PCA of RNA sequencing data from intestinal samples of mice with administration of vehicle or HKLJ. (B) Heatmap of the 1177 differential genes from ALD mice treated with or without HKLJ. (C) GSEA plots showed a positive correlation between the tight junction pathway and HKLJ treatment based on the RNA-seq data. The heatmap displayed the genes that contributed the most to the enrichment score. (D) FPKM of differential genes *Reg3b* and *Reg3g*. Four biological replicate numbers in each group. Statistical significance was determined using unpaired Student's two-tailed *t* test, and data represent the mean ± s.e.m., unless otherwise indicated.

**
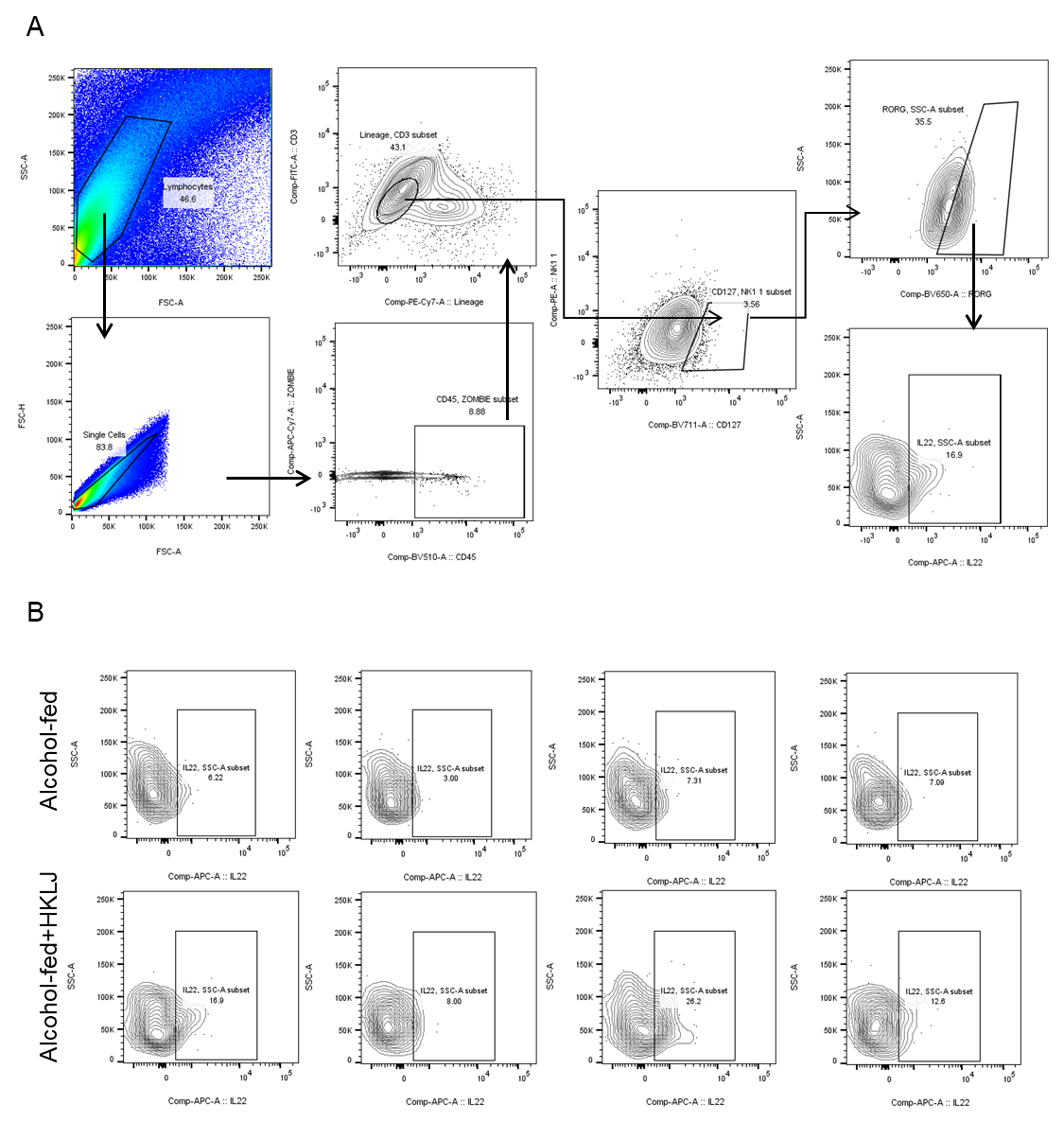
**

**Figure S8**: **Flow analysis of group 3 innate lymphoid cells (ILC3s).**

(A) The gating strategy for ILC3s was displayed (including removing adhesion cells, removing dead cells, and gating CD45^+^ Lineage^-^ CD3^-^ CD127^+^ RORγT^+^ cells). (B) IL-22^+^ ILC3s gated in ILC3s.

**
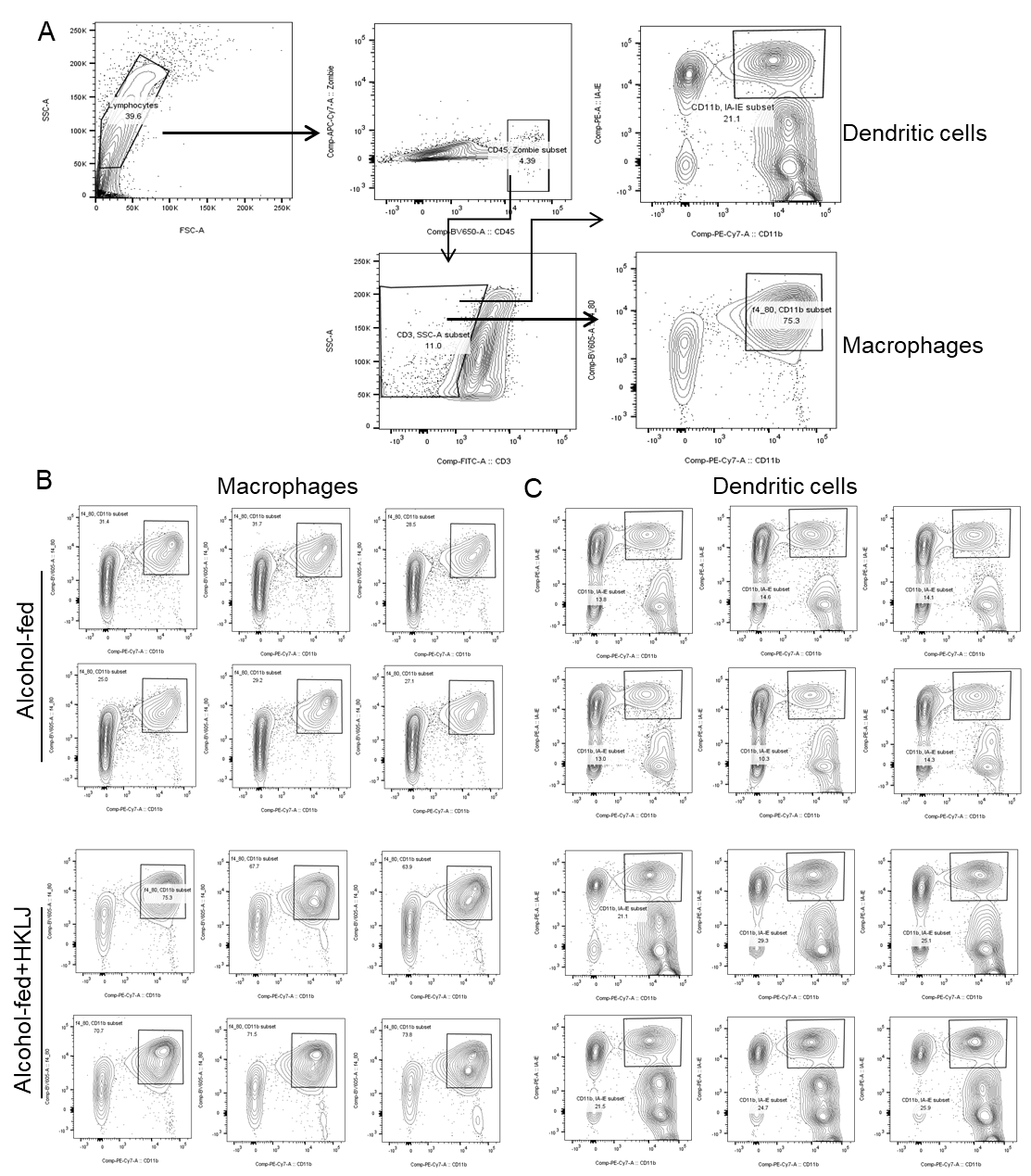
 Figure S9**: **Flow analysis of myeloid cells.**

(A) The gating strategies for macrophages and dendritic cells (DCs) were displayed (including removing dead cells, and gating CD45^+^ CD3^-^ cells). (B) F4/80^+^ CD11b^+^ macrophages gated in CD45^+^ CD3^-^ cells. (C) IA/IE (MHC II)^+^ CD11b^+^ DCs gated in CD45^+^ CD3^-^ cells.

**References**

[1] P. Xiu, R. Liu, D. Zhang, C. Sun, *BIO-PROTOCOL* **2018**, *8*.

[2] J. Gao, X. Zhao, S. Hu, Z. Huang, M. Hu, S. Jin, B. Lu, K. Sun, Z. Wang, J. Fu, R. K. Weersma, X. He, H. Zhou, *Cell Host & Microbe* **2022**, *30*, 1435.

[3] Y. Ge, M. Zadeh, M. Mohamadzadeh, *STAR Protocols* **2023**, *4*, 101936.
